# Specific mutations in H5N1 mainly impact the magnitude and velocity of the host response in mice

**DOI:** 10.1186/1752-0509-7-69

**Published:** 2013-07-29

**Authors:** Nicolas Tchitchek, Amie J Eisfeld, Jennifer Tisoncik-Go, Laurence Josset, Lisa E Gralinski, Christophe Bécavin, Susan C Tilton, Bobbie-Jo Webb-Robertson, Martin T Ferris, Allison L Totura, Chengjun Li, Gabriele Neumann, Thomas O Metz, Richard D Smith, Katrina M Waters, Ralph Baric, Yoshihiro Kawaoka, Michael G Katze

**Affiliations:** 1 Department of Microbiology, University of Washington, Seattle, WA 98195 USA; 2 School of Veterinary Medicine, Department of Pathobiological Sciences, Influenza Research Institute, University of Wisconsin-Madison, Madison, WI, USA; 3 Department of Microbiology and Immunology, School of Medicine, University of North Carolina at Chapel Hill, Chapel Hill, NC, USA; 4 Unité des Interactions Bactéries-Cellules, Institut Pasteur, 75015 Paris, France; 5 Biological Sciences Division, Pacific Northwest National Laboratory, Richland, WA, USA; 6 Washington National Primate Research Center, University of Washington, Seattle, WA, USA

**Keywords:** Influenza, Host, Response, Kinetics, Magnitude, Velocity, Transcriptomics, Proteomics, Multidimensional, Scaling

## Abstract

**Background:**

Influenza infection causes respiratory disease that can lead to death. The complex interplay between virus-encoded and host-specific pathogenicity regulators – and the relative contributions of each toward viral pathogenicity – is not well-understood.

**Results:**

By analyzing a collection of lung samples from mice infected by A/Vietnam/1203/2004 (H5N1; VN1203), we characterized a signature of transcripts and proteins associated with the kinetics of the host response. Using a new geometrical representation method and two criteria, we show that inoculation concentrations and four specific mutations in VN1203 mainly impact the magnitude and velocity of the host response kinetics, rather than specific sets of up- and down- regulated genes. We observed analogous kinetic effects using lung samples from mice infected with A/California/04/2009 (H1N1), and we show that these effects correlate with morbidity and viral titer.

**Conclusions:**

We have demonstrated the importance of the kinetics of the host response to H5N1 pathogenesis and its relationship with clinical disease severity and virus replication. These kinetic properties imply that time-matched comparisons of ‘omics profiles to viral infections give limited views to differentiate host-responses. Moreover, these results demonstrate that a fast activation of the host-response at the earliest time points post-infection is critical for protective mechanisms against fast replicating viruses.

## Background

Highly pathogenic H5N1 avian influenza (HPAI) viruses cause rare but severe disease in humans, with a case fatality rate approaching 60% among laboratory-confirmed cases. Although direct human-to-human transmission of these viruses does not occur, recent evidence suggests that only a few molecular changes in the viral surface hemagglutinin (HA) protein are sufficient to convert a non-transmissible HPAI virus into one capable of droplet transmission in the ferret model [1-3]. These findings raise concern over the possibility of a HPAI virus pandemic, and underscore the critical need for a better understanding of the mechanisms that control HPAI virus pathogenicity. Furthermore, the molecular dynamics of the host response and the complex interplay between virus-encoded determinants, host regulatory factors, H5N1 pathogenesis and severe lung disease is not well understood. Such information is essential for the development of more effective intervention strategies aimed at ameliorating human disease and loss of human life resulting from HPAI virus infections.

To unravel this interplay, we performed a systematic characterization of the global host response at the transcript and protein levels in lungs of mice infected by a panel of viruses that vary in pathogenicity, using a range of inoculation dosages. We also report a systematic collection of several corresponding phenotypic variables, including mouse body weights, lung virus titers, viral messenger RNA, and viral genomic RNA for each infected animal. Our panel of viruses included the A/Vietnam/1203/2004 [H5N1] wild-type virus (VN1203-WT), the lower pathogenicity A/California/04/2009 [H1N1] wild-type virus (CA04-WT), two previously described VN1203 mutant viruses – VN1203-HAavir [2] and VN1203-PB2-627E [4] – and two newly generated VN1203 mutant viruses – VN1203-NS1trunc and H5N1 VN1203-PB1F2del.

The H5N1 VN1203-WT virus is extremely virulent in mice [5], and elicits a host response that contributes in part to its pathogenicity [6]. The H5N1 VN1203-HAavir mutant virus harbors an altered multi-basic cleavage site – a virulence factor important for expanded tissue range [7-9] – and exhibits restricted systemic viral spread due to limited HA susceptibility to furin protease activity. The H5N1 VN1203-PB2627E mutant possesses an amino acid substitution (Lys-to-Glu) at position 627 in the PB2 polymerase subunit. This mutation is known to confer increased polymerase activity in mammalian cells [10], and also modulates anti-viral activity, apoptosis, and viral clearance [11]. Our newly generated H5N1 VN1203-NS1trunc mutant virus produces a 91 amino acid C-terminal truncation in the effector domain of the NS1 host response antagonist protein. The NS1 protein inhibits RIG-I activation [12] and cellular mRNA processing [13], and also promotes PI3K activation [13]. The truncation results in the loss of the NS1 nuclear localization signal, a PI3K-binding motif, and binding domains that support interactions with the cellular nuclear proteins *CPSF* and poly (A)-binding protein II (*PABII*) – two factors that function in the 3’-end-processing of cellular pre-mRNAs [14,15]. The newly generated H5N1 VN1203-PB1F2del mutant lacks expression of the PB1-F2 protein, potentially impacting an array of functions. PB1-F2 is a viral pathogenicity factor in mammals and birds [16], and has been shown to modulate viral polymerase activity [17,18], enhance lung inflammation [19], modulate innate immune responses [20,21], and demonstrate pro-apoptotic activity [14]. Finally, the CA04-WT virus, which is an H1N1 isolate from the 2009 pandemic season, induces lower pathogenicity in mice relative to VN1203 [5,22].

The extensive systematic analysis of transcriptomic and proteomic pulmonary responses to wild-type and mutant viruses we report here provides an unprecedented opportunity to assess the effect of specific virus attenuating mutations on global host responses *in vivo*, as well as the opportunity to directly examine the effects of dosage on the elicited host response. Here, we sought to specifically address how the kinetics of the host responses to the different viral infections differs, and how this kinetics is related to the outcome of infection. To address this, we used a systems biology approach to analyze this dataset, and we developed a new geometrical representation method and two criteria – the magnitude and velocity coefficients – to visualize and quantify the kinetics of the host response to the different viral infections. Using this approach, we have established that it is the magnitude and velocity of the early host response, rather than engagement of specific biological pathways per se, which mainly contributes to the observed pathogenicity of influenza viruses. Importantly, we show that the molecular kinetic of the host response was associated with clinical disease severity and virus replication.

## Results

### Assembly of phenotypic variables and lung transcriptomic and proteomic profiles from mice infected with low and high pathogenicity influenza viruses

We collected 230 transcriptomic and 198 proteomic profiles from the lungs of 20–week old C57BL/6 mice infected with CA04-WT, VN1203-WT, VN1203-HAavir, VN1203-PB2627E, VN1203-NS1trunc, or VN1203-PB1F2del. Mice were infected with a range of inoculation dosages (from 10^2^ to 10^6^ Plaque Forming Unit – PFU) and lungs were harvested at 1, 2, 4 and 7 days post-infection (dpi) to measure gene expression using oligonucleotide arrays and obtain proteomic profiles by liquid chromatography–mass spectrometry (LC-MS). Time-matched lung samples from mock-infected mice were collected and a total number of 300 transcriptomic profiles and 266 proteomic profiles are represented in our dataset.

Throughout this article, we use the following definitions to describe the different conditions: (i) *biological condition* refers to all samples infected by the same virus, at the same inoculation dose, and from the same dpi; (ii) *dosage condition* refers to samples infected by the same virus and at the same inoculation dose; (iii) *strain condition* refers to all samples infected by the same virus. Based on these criteria, the dataset is divided into 51 transcriptomic biological conditions and 42 proteomic biological conditions, representing 13 transcriptomic and 11 proteomic dosage conditions, and 6 strain conditions. Additional file 1: Figure S1 provides a schematic representation of the collected infected ‘omics profiles across the time course.

**Figure 1:**
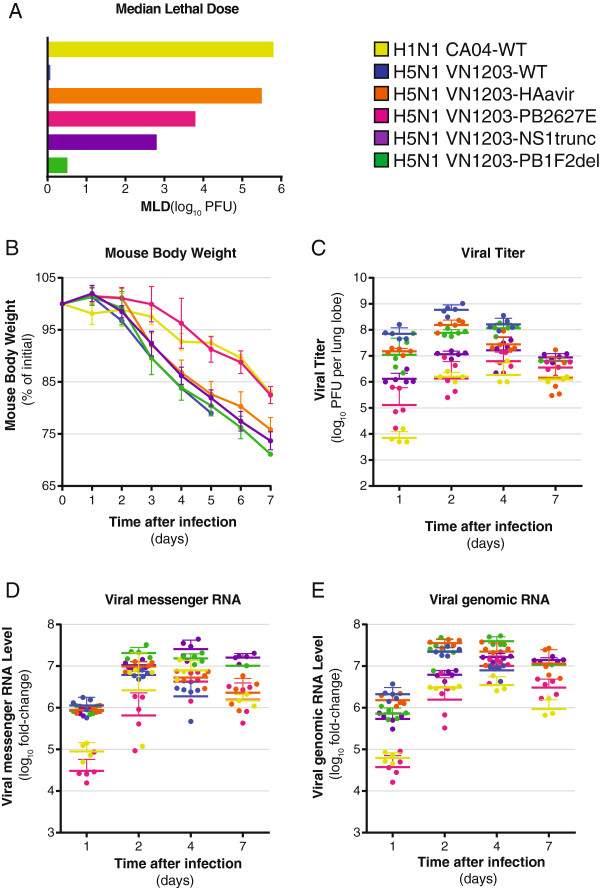
**MLD and phenotypical variables collected for all viruses at the 10**^**4 **^**PFU infection dosage. (A)** Median lethal dose (MLD) values of the different viruses in 6-week-old BALB/c mice. MLD values for VN1203 and mutants were performed in one experiment (3 mice per inoculation dosage), and the previously calculated MLD for CA04 is included for comparison [22]. **(B)** Mice body weight measurements collected each day from 1 to 7 days post-infection for the 10^4^ PFU infection dosages. For each virus and time point, the mean and the standard deviation of the mice body weight measurements are indicated by a vertical bar. **(C)**, **(D)** and **(E)** Viral titer, viral messenger RNA, and viral genomic RNA measurements collected at 1, 2, 4 and 7 days post-infection for the 10^4^ PFU infection dosages. For each virus and time point the set of individual measurements are indicated by filled dots and the mean amongst the individual samples is indicated by a horizontal bar. For the experiments shown in panels B-E, four to five mice were used for all VN1203-WT and pathogenicity mutant infections, while 3–4 mice were used for the CA04 infections. The same animals were used to derive all the phenotypical data (i.e. weight loss, virus titer and virus mRNA/genomic RNA levels).

The different viruses can be ranked based on their pathogenicity as determined by Median Lethal Dose (MLD) values in 6-week-old BALB/c mice (Figure 1A). The VN1203-WT (MLD < 1 PFU) and the VN1203-PB1F2del (MLD = 3.2 PFU) viruses were associated with the highest level of pathogenicity. The CA04-WT (MLD = 630,957 PFU; previously determined [22]) and the VN1203-HAavir (MLD = 316,228 PFU) viruses were associated with the lowest level of pathogenicity. The VN1203-NS1trunc (MLD = 631 PFU) and the VN1203-PB2627E (MLD = 6,310 PFU) viruses showed an intermediate level of pathogenicity.

Morbidity associated with infection, determined by mouse body weight loss (Figure 1B), viral replication quantified by viral titration (Figure 1C), viral messenger RNA (Figure 1D), and viral genomic RNA (Figure 1E) measured by quantitative RT-PCR were used to characterize the viruses in our study. These data were collected in the same experiment in which mouse lung tissues were collected for global transcriptome and proteome profiling. Over the 7-day time course, VN1203-WT and all of the mutant viruses – with the exception of VN1203-PB2627E – exhibited similar, rapid weight loss culminating in a 20-30% reduction from initial body weights by 5–7 days post-infection (Figure 1B). In contrast, both VN1203-PB2627E and CA04-WT displayed a more reduced rate of weight loss, which peaked at ~17% on day 7 post-infection. None of the mice survived to day 7 for the VN1203-WT infection at 10^4^ PFU. Virus titers were roughly distributed from low to high according to virus pathogenicity, with CA04-WT exhibiting the lowest mean titer in all four time-points and VN1203-WT exhibiting the highest (Figure 1C; see Additional file 2: Figure S2 for statistical comparisons). The one exception was titers observed in VN1203-HAavir infections, which rivaled that of VN1203-WT in all time-points. Both viral messenger RNA and genomic RNA production levels exhibited early (day 1) segregation into two groups, with VN1203-PB2627E and WT-CA04 displaying a 1–1.5 log_10_–fold reduction in expression compared to all the other viruses (Figure 1D and E and Additional file 2: Figure S2). Viral RNA expression levels remained partially segregated on day 2, and by day 4, expression levels were more similar for all viruses in the panel.

In brief, influenza viruses used in this study exhibited a range of lethality, morbidity, and replication in mice. Consistent with our expectations, the VN1203-WT virus was the most virulent, with a very low MLD, the most rapid weight loss, and the highest virus replication at early dpi (day 1 and 2).

### Gene magnitude changes significantly distinguish the strain and biological conditions

Deletion of PB1-F2, K627E substitution in PB2, elimination of the HA multi-basic cleavage site, and truncation of NS1 all decreased VN1203 virulence in mice, and CA04-WT shows the lowest virulence in mice. We first aimed to determine whether the attenuated phenotype for each virus was the result of a different host response.

**Figure 2:**
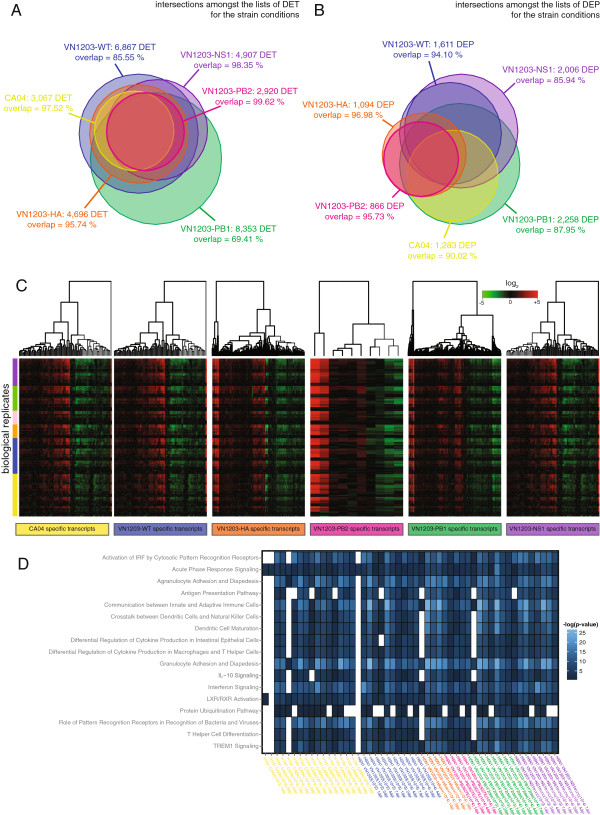
**Intersections between the lists of DET and DEP for the strain conditions. (A)** and **(B)** Proportional Euler-diagrams showing the intersections between the lists of differentially expressed transcripts and proteins identified for the strain conditions. Proportional Euler-diagrams visually represent the cardinalities of sets and intersection sets of differentially expressed transcripts or proteins by area-proportional circle graphics. Each list of DE transcripts or proteins is then represented by a circle with a diameter proportional to the cardinality of the list and the overlaps between the circles are proportional to the cardinality of the intersections between the lists. For each strain condition the number of transcripts or proteins found as DE in the host response is indicated as well as the degree of overlap — quantified as the percentage of transcripts or proteins also found as differentially expressed in another condition. **(C)** Heatmap of the transcriptomic expression values, ratioed to mock-infected samples, for each infected sample. The heatmaps have been restricted to the lists of transcripts found as specific for each viral strain and the different subsets of transcripts specific are indicated. For each set of specific transcripts, hierarchical clustering have been performed and represented using dendrograms. Biological samples have been ordered by strain conditions, sorted by inoculation concentrations and then by increasing days post-infection. **(D)** Heatmaps showing the statistical over-representation of the canonical pathways based on the lists of transcripts found as differentially expressed (compared to the mock-infected conditions) for each of the 51 biological conditions. This heatmap has been restricted to only display the top canonical pathways over-represented across all the dataset.

For each biological condition, differentially expressed transcripts (DET) and proteins (DEP) compared to the mock-infected samples were identified and the overlap between the lists of DET or DEP of each virus were compared. Figure 2A and B provide visual representations of the intersections between the lists of DET and DEP for the strain conditions using proportional Euler-diagrams [23]. There were large degrees of overlap (defined here as the percentage of DET or DEP also found in another condition) between the strain conditions, between 69.41% - 99.62% and 85.94% - 96.98%, DET and DEP, respectively. The VN1203-PB1F2del strain condition had the smallest degree of overlap at the transcriptomic (69.41%) level, and a relatively small degree at the proteomic (87.95%) levels. Notably, at 7 dpi, only one transcriptomic profile was available for the VN1203-PB1F2del strain condition inoculated at the 10^4^ PFU concentration. Hence, with only one sample available, the statistical identification of DET lead to the identification of a fraction of false-positive, explaining this lowest degree of overlap observed for this strain condition at the transcriptomic level. The VN1203-WT strain condition also showed a relatively small degree of overlap at the transcriptomic (85.55%) level; and the VN1203-NS1trunc strain condition showed the smallest degree of overlap at the proteomic (85.94%) level compared to the other strain conditions. It should be noted that the strain conditions with the lowest degrees of overlap corresponded to the same strain conditions that triggered the largest amount of DET and DEP. Similar large amounts of overlap have been identified between the dosage conditions, with degrees of overlap ranging from 68% to 98.81% for the transcriptome and from 89.87% to 99.89% for the proteome. Despite differences in clinical manifestation of the disease, these results suggest that the specific mutations in VN1203 examined herein resulted in the induction of similar groups of transcripts and proteins compared to VN1203-WT, implying that magnitude and/or the kinetics of dysregulation of these overlapping genes might differentiate the viruses. Figure 2C is a heatmap of the transcript expression values, ratioed to the mock-infected samples, of each infected sample of our dataset. This heatmap has been restricted to the lists of transcripts specific to each strain condition (i.e. transcripts found as differentially expressed within one viral strain but not in the others). We cannot distinguish any particular patterns or sets of transcript specific to any viral strain based on these transcript expression values. The transcript or protein subsets specific to each viral strain are the consequences of small variations in the host response, detected by the statistical procedures, that have no specific biological meaning.

Functional enrichment analysis of the biological conditions also indicated a large degree of overlap in the host response – at least qualitatively – irrespective of the virus used to infect the mice. For instance, Acute Phase Response Signaling, Antigen Presentation Pathway, LXR/RXR Activation, and Protein Ubiquitination were pathways universally enriched (Figure 2D). Differences between the biological conditions were mainly explained by different degrees of enrichment scores, smaller for the early time-points post-infection and larger for the late time-points post-infection. Similarly, within each viral strain, the enrichment scores were smaller for the lower infection concentrations and larger for the higher infection concentrations. Overall, the qualitative similarity in the host transcriptomic and proteomic response to various influenza viruses suggested that the different viruses stimulate activation of common host response signaling mechanisms with different degrees of magnitude.

Altogether, these results demonstrate that it is mainly the magnitude of expression changes within the same transcripts or proteins, rather than the induction of different gene sets, that differentiate the host response to CA04-WT, VN1203-WT, and H5N1 VN1203 mutants. Thus, isogenic viruses and different inoculation dosages likely trigger the same differentially regulated genes, but with different degrees of magnitude at both the transcriptomic and proteomic levels. This analysis was performed on the transcriptomic and proteomic levels independently, and in parallel. Though we noted this magnitude effect was stronger in the proteomic dataset compared to the transcriptomic dataset, we found the same conclusions in each separate analysis.

### The kinetics of the host response to VN1203-WT is captured by 3 main eigentranscripts and 3 main eigenproteins

To better characterize the host responses, and to be able to quantify their kinetics, we used a Singular Value Decomposition analysis to capture and cluster transcripts and proteins associated with the system’s dynamics, as described in [24]. Transcripts and proteins previously identified as differentially expressed in at least one biological condition that correlated with the eigentranscripts or eigenproteins (right-singular vectors of the singular value decomposition) were identified using the Pearson’s coefficient, with eigentranscripts or eigenproteins representing significant patterns of expression profiles, associated with the kinetics of the system. Transcripts and proteins that did not correlate with any eigentranscript or eigenprotein, respectively, were not considered as significantly associated in the dynamics of the host response (Pearson’s coefficient cutoff = 0.65). This approach was used to examine the host response to VN1203-WT, independently of the inoculation dosage, that was then subsequently used as a reference to compare the VN1203 mutants and CA04-WT. Mock infected biological conditions were not used in the inference of eigentranscripts and eigenproteins, and biological conditions at 1 dpi and 2 dpi were not used in the inference of the eigenproteins because of the relatively low levels of proteins detected as differentially expressed at these time-points.

**Figure 3:**
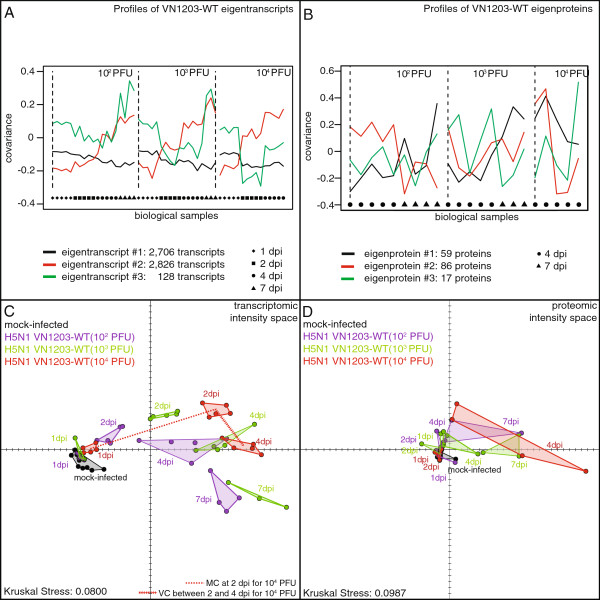
**Eigengenes in the kinetics of the VN1203-WT response, and MDS representations of the infected samples. (A)** and **(B)** Profiles of the eigentranscripts and eigenproteins identified in dynamic of the host response to H5N1 VN1203 wild-type. Number of transcripts and proteins correlating with each eigentranscript and eigenprotein are indicated. All the individual shape represent the transcriptomic or proteomic profile of a mice lung infected by the VN1203 wild-type virus. Biological samples have been sorted by inoculation concentrations and then by increasing days post-infection. **(C)** and **(D)** Multidimensional Scaling representations of the transcriptomic and proteomic profiles of the H5N1 VN1203 wild-type infected samples. Each dot in the representations is the transcriptomic or proteomic profile of a biological sample plotted in the intensity space of expression signals. Pairwise distances between the dots are proportional to the transcriptomic or proteomic distances between the samples. Transcriptomic and proteomic distances have been calculated based on the signature of 5,660 transcripts and 162 proteins that significantly correlate with one eigentranscript or eigenprotein. Dots are colored in order to indicate the dosage conditions, and biological conditions are indicated by the convex hull of the set of biological replicates and labeled to indicate the time point post-infection. The Kruskal Stress shown in each representation quantifies the quality of the geometrical representation as a fraction of the information lost during the dimensionality reduction procedure. Schematic projections of the Magnitude and Velocity Coefficients are illustrated at 10^4^ PFU in the transcriptomic MDS representation. The Magnitude Coefficient MC at 2 dpi – quantifying the transcriptomic distance between mocks and 2 dpi samples – is illustrated by a dashed red line, and the Velocity Coefficient (VC) at 4 dpi – measuring the velocity between 4 and 2 dpi samples divided by time – is illustrated by an arrow dashed red line.

We found a total of 5,660 transcripts that correlated with 3 main eigentranscripts (eigentranscript #1 contained 2,706 transcripts, eigentranscript #2 contained 2,826 transcripts, and eigentranscript #3 contained 128 transcripts). There were a total of 162 proteins that correlated with 3 main eigenproteins (eigenprotein #1 contained 59 proteins, eigenprotein #2 contained 86 proteins, and eigenprotein #3 contained 17 proteins). Additional file 3: Table S1 and Additional file 4: Table S2 provide the lists of transcripts and proteins associated with each eigentranscript and eigenprotein, respectively. The patterns of the eigentranscripts and eigenproteins identified in the VN1203-WT host response are represented in Figure 3A and B. Eigentranscript #2 was increasing over time, whereas eigentranscript #1 was decreasing over time. Eigentranscript #3 showed a distinct pattern with a transient decrease at 2 and 4 dpi, followed by an increase at 7 dpi. Eigenprotein #1 was increased in abundance over time. In brief, the dynamics of the transcriptomic and proteomic host response were captured by 3 sets of co-expressed transcripts and 3 sets of co-expressed proteins with different patterns of variations.

For each set of transcripts and proteins, we identified over-represented biological functions, canonical pathways, and upstream regulators (Tables 1 and 2). Eigentranscript #2 profile was enriched for transcripts associated with Death Receptor Signaling, Role of Pattern Recognition Receptors in Recognition of Bacteria and Viruses, and Apoptosis Signaling, as well as the Protein Ubiquitination Pathway, which has previously been observed in mouse lung responses following pandemic 1918 influenza virus infection [25]. There was representation of transcripts encoding immuno proteasome components (e.g., *PSMB9* and *PSMB10*), ubiquitin-protein conjugates (e.g., *USP18* and *USP3*), and heat shock proteins (e.g., *HSPA5* and *HSPB7*) accompanying the Protein Ubiquitination Pathway. Eigentranscript #1 profile was comprised of transcripts predominantly associated with metabolic cellular processes including Glutathione-mediated Detoxification (e.g., *GSTK1* and *GSTA3*) and Xenobiotic Metabolism Signaling (e.g., *AHR* and *CAT*). Respiratory System Development and Function was an enriched biological function for eigentranscript #1 that included *RARG* and *RARB* transcripts, two ligand-dependent nuclear receptors that modulate an array of cellular responses related to cellular growth. Eigentranscript #3 profile was enriched for transcripts associated with T cell responses, *CD3G*, *ZAP70*, and *ICOS*, related to T Cell Receptor Signaling, iCOS-iCOSL Signaling in T Helper Cells, and Regulation of IL-2 Expression in Activated and Anergic T Lymphocytes. Activated CD8 T cells in the lungs of mice infected with H5N1 virus are impaired in their ability to control viral replication linked to high pathogenicity [26].

**Table 1:** Functional enrichment of the eigentranscript identified in the host response kinetics to VN1203

**Eigentranscript (No. of molecules)**	**Bio functions category (functions annotation *p*-value range)**	**Canonical pathway ( *p *-value)**	**Upstream regulator ( *p *-value of overlap)**
***eigentranscript #1*** (2706)	Embryonic development (5.55E-11–5.24E-03)	Axonal guidance signaling (8.71E-07)	NKX2-1 (5.35E-13)
	Organ development (5.55E-11–5.24E-03)	Basal cell carcinoma signaling (1.82E-05)	PPP3R1 (2.10E-11)
	Organismal development (5.55E-11–5.24E-03)	Glutathione-mediated detoxification (6.61E-05)	DMD (1.36E-05)
	Tissue development (5.55E-11–5.24E-03)	Xenobiotic metabolism signaling (2.19E-04)	BMP6 (6.45E-05)
	Respiratory system development and function (1.19E-10–2.38E-03)	Tryptophan degradation (4.68E-04)	GLI1 (1.38E-04)
***eigentranscript #2*** (2826)	Cellular function and maintenance (3.38E-55–2.01E-09)	Death receptor signaling (5.01E-09)	LPS^*^ (9.74E-111)
	Cellular function and maintenance (3.38E-55–2.01E-09)	Apoptosis signaling (7.94E-09)	IFNG (2.42E-87)
	Hematological system development and function (1.34E-51–3.31E-09)	Role of pattern recognition receptors in recognition of bacteria and viruses (1.78E-08)	TNF (8.84E-65)
	Tissue morphology (5.34E-51–2.48E-10)	Induction of apoptosis by HIV1 (4.07E-08)	IL1B (1.15E-56)
	Cellular growth and proliferation (5.55E-11–5.24E-03)	Protein ubiquitination pathway (4.17E-08)	TP53 (6.72E-55)
***eigentranscript #3*** (128)	Cell cycle (1.67E-11–1.60E-02)	Pyrimidine deoxyribonucleotides de novo biosynthesis I (5.13E-05)	E2F4 (5.42E-12)
	Cell cycle (1.67E-11–1.60E-02)	T Cell receptor signaling (1.70E-04)	FOXM1 (1.84E-11)
	Cellular development (5.83E-10–1.60E-02)	iCOS-iCOSL signaling in T helper cells (2.69E-04)	CDK4 (1.44E-10)
	Hematological system development and function (5.83E-10–1.60E-02)	Mitotic roles of polo-like kinase (4.47E-04)	CDKN1A (1.09E-09)
	Hematopoiesis (5.83E-10–1.60E-02)	Regulation of IL-2 expression in activated and anergic T lymphocytes (8.51E-04)	CCND1 (1.66E-09)

For each eigentranscript identified in the kinetics of the host response to H5N1 VN1203 wild-type, the number of correlating transcripts is indicated. For each set of transcript the top 5 associated over-represented biological functions, canonical pathways, and upstream regulators are indicated. Ingenuity pathway analysis was used to determine the top 5 bio function categories, canonical pathways, and upstream regulators. The functions annotation p-value range represents the range of p-values for the functions annotations associated with each bio function category. The p-value of overlap associated for each upstream regulator indicates the significance of the overlap between the genes targeted by the upstream regulator in the IPKB database and the experimental dataset. *Upstream regulators with an apteryx signify a chemical reagent or chemical drug.

**Table 2:** Functional enrichment of the eigenproteins identified in the host response kinetics to VN1203

**Eigenprotein (No. of molecules)**	**Bio functions category (functions annotation *p*-value range)**	**Canonical pathway (*p***-**value)**	**Upstream regulator (*p***-**value of overlap)**
***eigenprotein #1*** (59)	Free radical scavenging (8.24E-07–2.56E-02)	NRF2-mediated oxidative stress response (2.40E-05)	D3T^*^ (2.77E-06)
	Drug metabolism (3.50E-05–2.87E-02)	Glutathione-mediated detoxification (2.69E-04)	MAPT (6.72E-06)
	Auditory disease (6.12E-05–6.12E-05)	G Beta gamma signaling (2.82E-04)	TP53 (8.41E-06)
	Developmental disorder (1.52E-04–2.56E-02)	Ceramide signaling (2.45E-03)	NFE2L2 (1.40E-05)
	Hereditary disorder (1.52E-04–6.45E-03)	EIF2 signalin (3.02E-03)	PSEN1 (1.16E-04)
***eigenprotein #2*** (86)	Neurological disease (3.89E-08–8.48E-03)	Acute phase response signaling (1.26E-25)	Nitrofurantoin^*^ (3.03E-12)
	Cell-to-cell signaling and interaction (8.90E-08–1.08E-02)	LXR/RXR Activation (1.58E-13)	Captopril^*^ (1.79E-08)
	Tissue development (8.90E-08–1.08E-02)	Clathrin-mediated endocytosis signaling (9.77E-08)	IL6 (1.83E-07)
	Metabolic disease (2.66E-07–1.02E-02)	Complement system (3.02E-07)	Gentamicin^*^ (5.04E-07)
	Lipid metabolism (5.24E-07–1.17E-02)	Coagulation system (3.55E-07)	T_3_^*^ (8.12E-07)
***eigenprotein #3*** (17)	Cell death and survival (1.82E-05–4.83E-02)	Breast cancer regulation by Stathmin1 (1.70E-05)	PLG (2.14E-04)
	Hematological system Development and function (9.90E-05–4.48E-02)	Cardiac β-adrenergic signaling (1.91E-04)	APP (1.19E-03)
	Organismal injury and abnormalities (1.63E-04–4.83E-02)	AMPK signaling (2.24E-04)	PSEN1 (2.06E-03)
	Tissue morphology (8.38E-04–4.19E-02)	CREB signaling in neurons (4.07E-04)	MAP2 (2.12E-03)
	Cancer (8.38E-04–4.99E-02)	Role of NFAT in cardiac hypertrophy (4.57E-07)	GNG7 (2.12E-03)

For each eigenprotein identified in the kinetics of the host response to H5N1 VN1203 wild-type, the number of correlating transcripts and proteins are indicated. For each set of proteins the top 5 associated over-represented biological functions, canonical pathways, and upstream regulators are indicated. Ingenuity pathway analysis was used to determine the top 5 bio function categories, canonical pathways, and upstream regulators. The functions annotation p-value range represents the range of p-values for the functions annotations associated with each bio function category. The p-value of overlap associated for each upstream regulator indicates the significance of the overlap between the genes targeted by the upstream regulator in the IPKB database and the experimental dataset. *Upstream regulators with an apteryx signify a chemical reagent or chemical drug.

Eigenprotein #2 profile had strong representation of proteins associated with Acute Phase Response Signaling (e.g., *SAA2* and *SERPINA1*), LXR/RXR Activation (e.g., *APOA1* and *APOA2*), Complement System (e.g., *CFB* and *C4B*) and Coagulation System (e.g., *F2* and *FGG*), cellular responses that are critical to host antiviral defenses. Examination of proteins associated with eigenprotein #1 profile showed enrichment of proteins related to modification of reactive oxygen species. For example, there was enrichment of *PRDX1*, *PTPLAD1*, and *ERP29*, suggesting heightened oxidative stress responses in the lungs of VN1203-infected mice. H5N1 infection causes acute lung injury and mice inoculated with inactivated H5N1 virus have compromised lung function, including altered lung elastance and increased ROS and oxidized phospholipid production in the lung [27]. Represented within eigenprotein #3 profile, were *APOH* and *PLG* proteins involved in opsonization of cells, as well as the NADH dehydrogenase components *NDUFA11* and *NDUFA13*.

In conclusion, the three sets of co-expressed transcripts identified in response to VN1203-WT were associated with immune and apoptosis signaling pathways that increased over time, metabolic cellular processes that were largely decreasing, and T cell signaling pathways that exhibited a biphasic pattern. The three sets of proteins capturing the host response dynamics were related to host antiviral defenses, reactive oxygen species observed to increase as infection progressed, and opsonization. Notably, upstream regulators highly enriched in each of the transcript or protein sets were identified (p-value as low as 10^-111^ for LPS regulating eigentranscript #2), which confirmed that this method captures groups of co-regulated transcripts/proteins. Importantly, there was an overlap of 29 signaling pathways that were found in both transcript and protein sets, including Role of NFAT in Regulation of the Immune Response, Apoptosis Signaling and Coagulation System, suggesting complementary sensitivity between the transcriptomic and proteomic profiles.

### Different doses of VN1203 WT impact the magnitude and velocity of the pulmonary response to infection

To determine the effects of increasing dosage on the kinetics of the host response to VN1203-WT, we used Multidimensional Scaling (MDS) [28,29] as a visualization method and introduced two new criteria quantifying magnitude and velocity changes in the signature (Figure 3C and D). Each dot in the MDS representations is the transcriptomic or proteomic profile of a biological sample, and pairwise distances between dots are proportional to the transcriptomic or proteomic distances (Euclidean distances) between the samples. The MDS representation, calculated using the sets of transcripts and proteins associated with the VN1203-WT kinetics, highlighted interesting differences between the dosage conditions. While all samples from different dosages were close to the mocks at 1 dpi, the late time-points followed a similar curved trajectory. Different magnitudes, defined as the distance of one biological condition to the mock-infected condition, and velocities, defined as the speed of the host response moving from one time point to the next time point, were observed for the three dosage conditions for both the transcriptomic and proteomic levels. For example, infection with the highest dose of VN1203-WT virus (10^4^ PFU) induced a similar change in host response at 4 dpi as VN1203-WT infection with the lowest dose (10^2^ PFU) observed 3 days later at 7 dpi (Figure 3C). Compared to the transcriptomic profiles, the MDS representation of the proteomic profiles showed more noise in the data (Figure 3D). For instance, we observed that the variations within the groups were as high as the variations between groups. The proteomic samples collected at 1 and 2 days post-infection samples were closely clustered with the mocks. Although we were unable to differentiate these samples, we observed a similar effect of dose on the trajectories at 4 and 7 dpi, with the highest dose reaching the end of the trajectory more quickly compared to the lower doses. As such, the biological interpretations derived from the proteomic analysis have to be taken into careful considerations.

**Figure 4:**
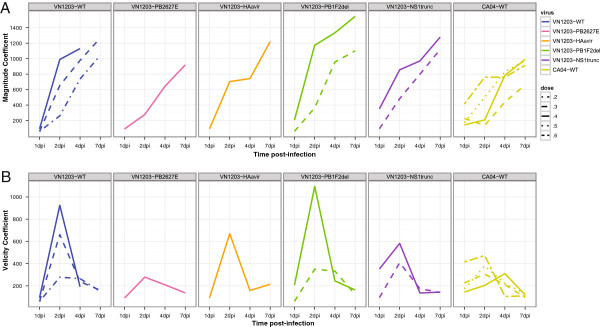
**Profiles of Magnitude Coefficients and Velocity Coefficients for the different viruses and dosage conditions. (A)** Variation of Magnitude Coefficients over time for the different viruses. **(B)** Variation of Velocity Coefficients over time for the different viruses. Profiles are colored by viral strain and line types represent the different doses. The Magnitude Coefficient (MC) quantifies the magnitude effect as the transcriptomic or proteomic distance from one biological condition to the matched mock-infected condition. The Velocity Coefficient (VC) quantifies the velocity effect as the speed of the transcriptomic host response to move from one time point to the next one. Both the MC and VC were calculated based on the centroids of the biological conditions and the transcriptomic or proteomic distances are calculated based on the lists of transcripts associated with the kinetics of the host response to VN1203 wild-type.

In order to quantify these magnitude and velocity effects in the kinetics of the host response, we defined two criteria, the Magnitude Coefficient (MC) and the Velocity Coefficient (VC). The MC quantifies the magnitude effect as the transcriptomic distance from one biological condition to the matched mock-infected condition. The VC quantifies the velocity effect as the speed of the transcriptomic host response moving from one time point to the next in the succession of infection. Both the MC and VC were calculated based on the centroids (arithmetic means) of the biological conditions and the transcriptomic distances (Euclidean distances) based on the signatures of transcripts associated with the kinetics of the host response to VN1203-WT (Additional file 5: Table S3, Figure 4). MC and VC were calculated only for the transcriptomic data, as we were unable to discriminate proteomic samples at early time-points (1 and 2 dpi), resulting in a lack of sensitivity for the analysis. We first observed that increasing the inoculation dose was related to an increase in magnitude of the host response. Indeed, at each dpi, infection with 10^2^ PFU triggered a lower MC compared to 10^3^ PFU, while 10^4^ PFU triggered the strongest MC. Moreover, different inoculation dosages also resulted in different velocities of the host response changes. With regards to time, the greatest transcriptomic VC difference was found at 2 dpi, followed by the difference quantified between 1 and 2 dpi. With regards to dosage, the lowest transcriptomic VC coefficient difference was found for 10^2^ PFU and the greatest transcriptomic VC coefficient difference was found for 10^4^ PFU.

These results show that a similar kinetic signature of 5,660 transcripts and 162 proteins is dysregulated after infection with VN1203-WT at different doses, and that increasing dosages lead to higher magnitude changes of these transcripts/proteins and an increased velocity, especially between 1 and 2 dpi.

### Mutations in VN1203 impact the magnitude and velocity of the host response

**Figure 5:**
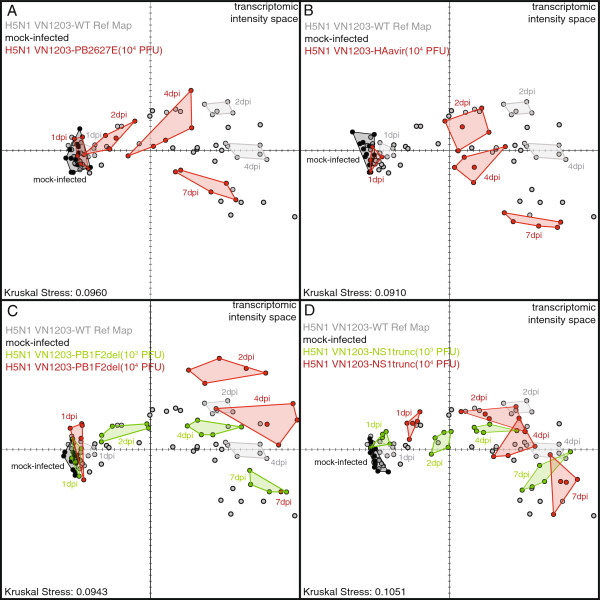
**MDS Projections of the VN1203 mutants infected transcriptomic profiles over the VN1203-WT Reference Map. (A)** Multidimensional Scaling Projection (MDS Projection) of the transcriptomic profiles of the VN1203-PB2627E dosage condition over the VN1203 wild-type Multidimensional Scaling Reference Map (MDS Ref Map). **(B)** MDS Projection of the transcriptomic profiles of the VN1203-HAavir dosage condition over the VN1203 wild-type MDS Ref Map. **(C)** MDS Projection of the transcriptomic profiles of the VN1203-PB1F2del dosage conditions over the VN1203 wild-type MDS Ref Map. **(D)** MDS Projection of the transcriptomic profiles of the VN1203-NS1trunc dosage conditions over the VN1203 wild-type MDS Ref Map. Each dot is the transcriptomic profile of a biological sample plotted in the intensity space of gene expression. Pairwise distances between the dots are proportional to the transcriptomic distances between the samples. MDS Projections allow to project additional ‘omics profiles over a predefined MDS representation (MDS Ref Map). Transcriptomic distances have been calculated based on the signature of 5,660 transcripts that significantly correlate with one eigentranscript. Dots are colored in order to indicate the dosage conditions, and biological conditions are indicated by the convex hull (i.e. the smallest convex set containing the points [30]) of the set of biological replicates and labeled to indicate the time point post-infection. Samples and biological conditions of the H5N1 VN1203 wild-type 10^4^ PFU infection dosage are indicated by gray dots and gray convex hulls. Hence the grey spots that are connected represent the -omics profiles of mice lung infected by the VN1203 wild-type virus at 10^4^ PFU, while the ones not connected represent the -omics profiles for the other infection concentrations. The Kruskal Stress shown in each representation quantifies the quality of the geometrical representation as a fraction of the information lost during the dimensionality reduction procedure.

Having characterized VN1203-WT dynamics, we were now able to determine how each specific VN1203 mutant altered the kinetics of the host responses. In particular, our aim was to better quantify the magnitude differences between WT and mutants that were introduced with Figure 2. We next developed a new geometrical representation method in order to compare the kinetics of the host response to the various VN1203 mutants relative to the host response to VN1203-WT at the transcriptomic level. Using the MDS representation presented in Figure 3C as a reference, we projected the transcriptomic profiles of samples infected by the four VN1203 mutant viruses individually at 10^4^ PFU (Figure 5A-D) and, in the case of VN1203-NS1trunc and VN1203-PB1F2del, at 10^3^ and 10^4^ PFU dosages (Figure 5C and D). While traditional MDS methods project a set of high dimensional objects into a lower dimensional space for visualization purposes, the MDS method that we developed allows for projection of additional objects over a predefined MDS representation (see Methods section). Thus, the resulting representation allows us to visualize the similarities and differences between the WT and mutant samples, and MC and VC allow us to quantify the kinetic changes in the host response related to magnitude and velocity, as previously described for VN1203-WT. The reference representation is named a MDS Reference Map and the resulting projections are named MDS Projections. Quantification of magnitude and velocity revealed different information about each mutant.

For VN1203-PB2627E, both the MDS projection (Figure 5A) and the MC and VC profiles (Figure 4) for the 10^4^ dosage were similar to that observed for VN1203-WT at the 10^2^ dosage. Therefore, the main effect of the PB2-627E mutation on the host-response was to decrease it to a level similar to that induced by 100 times less VN1203-WT. This is consistent with the reduced replication we observed for VN1203-PB2627E in the current study (Figure 1B) and with a previous study showing that the main effect of the PB2-627E mutation in the VN1203 background was to reduce virus replication in the lung [5].

The main effect of VN1203-HAavir was also found to be an attenuated host response, with the VN1203-HAavir 10^4^ dosage exhibiting an MDS projection and VC/MC profiles similar to that observed for VN1203 WT at the 10^3^ dosage (Figures 4, and 5B). The only difference was that VC and MC at 4 dpi were slightly lower than the VN1203-WT, while no difference in weight loss or viral replication was observed between the two viruses at that dpi.

Strikingly, whereas VN1203-PB1F2del VC and MC profiles at 10^3^ PFU were intermediate between VN1203-WT 10^2^ and 10^3^, at the highest dose (10^4^ PFU), both the peak of VC at 2 dpi and the peak of MC at 7 dpi were higher than VN1203-WT at the same dose (Figures 4 and 5C). It should be noted that both body weight loss and virus replication were similar at this dose between the VN1203-WT and this mutant (Figure 1). Moreover, during the course of infection, four out of the five mice died between 4 and 7 dpi, indicating a high virulence of this mutant at 10^4^ PFU.

Finally, the MC and VC profiles associated with VN1203-NS1trunc mutation largely changed at early dpi (1 and 2 dpi). At 1 dpi, these 2 coefficients were higher in the mutant than in the matched dose of the WT, but lower at 2 dpi (Figures 4, and 5D). This was related to the enhanced, early expression (i.e. at 1 dpi, relative to 2 dpi for all other viruses) of transcripts directly involved in the innate immune response and inflammation (Additional file 6: Figure S3), which was also reported previously [31]. It was interesting to find that this behavior was very similar to the CA04 at the highest dose (10^6^ PFU). CA04-WT was used as a much lower pathogenicity comparator which confirmed that the main effect of increasing infection dose was to increase the magnitude of the host-response (Additional file 7: Figure S4, and Figure 4).

To summarize, each mutant of VN1203 dysregulated the kinetic signature of 5,660 transcripts with a specific dynamic in terms of both magnitude of changes (MC), and speed at which they occurred (VC). While VN1203-PB2E627 and HAavir induced changes similar to those induced by 100 or 10 times lower doses of VN1203-WT respectively, VN1203-PB1F2del had a bimodal response with attenuation at 10^3^ PFU, but an increase in both magnitude and velocity at 10^4^ PFU. Finally, VN1203-NS1trunc induced changes in the 5,660 transcripts with a kinetics and magnitude more similar to CA04-WT 10^6^ PFU than VN1203-WT.

### Magnitude and velocity of the host responses are associated with weight loss and viral titer

**Figure 6:**
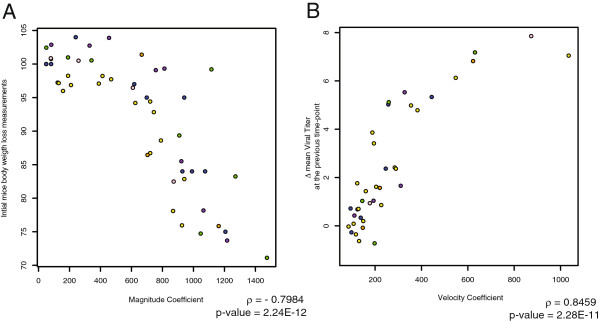
**Spearman’s coefficients between MC and body weight, and between VC and titer changes. (A)** Scatter plot between the Magnitude Coefficients and the mice body weight loss measurements. **(B)** Scatter plot between the Velocity Coefficients and the changes in viral titer measurements at the previous time-point. Each dot represents a couple of values for a single biological condition and is colored in order to indicate the viral strain according to the nomenclature of Figure 1. For each scatter plot the Spearman’s coefficient of correlation is indicated as well as the associated p-value.

To determine whether the VC and MC were related to disease outcome and viral pathogenicity, we performed a correlation analysis between these coefficients and the phenotypic variables. All biological conditions were considered together in these analyses to assess the correlations with a large significance. For each biological condition, we determined the mean body weight of the animals, viral titer, viral messenger RNA and viral genomic RNA measurements in the lung, and calculated the difference between the means of any two consecutive time-points for viral titer (Δ mean viral titer), viral RNA (Δ mean viral mRNA), and viral genomic RNA (Δ mean viral gRNA). For each transcriptomic biological condition the associated MC, VC, mean body weight, Δ mean viral titer, Δ mean viral mRNA, and Δ mean viral gRNA values are shown in Additional file 5: Table S3. A significant association was found between MC and mean body weight (Spearman’s rho = −0.7984), suggesting that the magnitude of host response is significantly associated with morbidity (Figure 6A). The VC was not significantly associated with any criteria when considering phenotypic variables within a given time-point; however, the VC was significantly associated with Δ mean viral titer (Spearman’s rho= 0.8459, Figure 6B), Δ mean viral mRNA (rho =0.8544), and Δ mean viral gRNA (rho =0.8426) at the previous time-point. This suggests that the velocity of the host response can be predicted by changes in viral replication at the preceding time-point.

In conclusion, the magnitude of changes in the host response of the 5,660 transcripts was correlated with morbidity at similar time-points, while the velocity of these changes between two time-points was associated with extent of changes in viral replication at previous time-points.

## Discussion

Highly pathogenic avian influenza H5N1 virus is known to induce aberrant host responses leading to severe immunopathology in the lung. In particular, the potent pro-inflammatory response, commonly referred to as ‘cytokine storm’, is the suspected cause of acute lung injury [32]. Genomic evidence from animal model systems shows HPAI H5N1 viruses strongly enhance cytokine and chemokine transcriptional responses compared to seasonal influenza viruses (reviewed in [33]) and 2009 pandemic H1N1 influenza viruses [34]. Here, we utilized a mouse model to study the system dynamics of H5N1 infection, comparing and contrasting the effects of increasing dosage and different previously described and newly generated viral mutants with varying degrees of pathogenicity toward the kinetics of pulmonary responses. Using a systems approach, we present a temporal analysis of transcriptomic and proteomic profiles measured from mouse lung samples collected during the acute phase of infection in order to better understand both dose-dependent and temporal mechanisms of host responses to influenza infection.

We report that the wild-type and mutant viruses differentially regulate a common set of transcripts and proteins, demonstrating a large degree of overlap in the host response among the different strain and dosage conditions. Regardless of the virus genetic change, we show that the host response is mainly impacted by the magnitude of gene expression and the speed at which these changes occur for this core set of transcripts, as opposed to transcriptomic differences explained by each specific mutation. We identified three main eigentranscripts associated with host immune mechanisms, including metabolic cellular processes and T cell signaling pathways, and three main eigenproteins associated with host antiviral defenses. Through a new geometric representation method and two criteria (the magnitude and velocity coefficients), we were able to visualize and quantify the magnitude and velocity kinetic effects of the host response to wild-type and mutant H5N1 VN1203 viruses, which were influenced by both the infection dosage and specific modifications to key H5N1 virulence determinants. Intriguingly, two newly generated H5N1 VN1203 mutant viruses, VN1203-PB1F2del and VN1203-NS1trunc, had the most distinct profiles, with apparent displacement relative to all other viruses at 2 dpi and 1 dpi, respectively. The divergent kinetic effects occurred mostly at early time-points (between days 1 and 2 post-infection), and we observed analogous kinetic effects with different doses of low pathogenicity 2009 pandemic H1N1 influenza virus (CA04-WT). Importantly, we demonstrate that the magnitude and velocity kinetic effects were associated with clinical disease severity and virus replication. Specifically, mouse weight loss correlated with the magnitude of the host response, and infectious viral particle production at a given time point correlated with the velocity of the host response at the next time-point.

Compared to the differentially expressed transcripts, changes in protein abundance were less apparent, due in part to inherent noise in the data that makes computational analyses of proteomic measurements particularly challenging [35,36]. However, we have been able to identify the same overall kinetic pattern, as observed with the transcriptomic data. Due to this variability, we have not used the proteomic data to quantify the magnitude and velocity effects and the biological interpretations we have drawn from the proteomic analysis have been done with careful consideration.

Previous work by Chang and coworkers, using a compendium of about 200 publicly available transcriptomic profiles of mouse lungs, showed differences in pathogenicity among respiratory viruses were explained in part by the changes in magnitude of gene expression [37]. However, due to constraints of the meta-analysis, time was not taken into consideration. Moreover, the different microarray platforms and mouse genetic backgrounds used in the different datasets introduced noise into the analysis. Here, we have expanded upon the findings by Chang et al. through a kinetic analysis that incorporates the dimension of time, uses a single mouse genetic background and isogenic respiratory viruses that differ based on mutations to known pathogenicity determinants. Transcriptomic and proteomic samples were collected in a systematic manner to generate a comprehensive dataset that provides a powerful resource for modeling pathogen-host interactions. The large sample number allows to infer co-expression and co-regulation networks for identification of unknown associations and dynamic interactions between biological components. Moreover, the extensive collection of sampled time-points allows to model causality of the biological system for discovery of novel biological events. In addition, a large panel of machine learning and data mining algorithms can be used, trained and tested based on this assembled dataset. While our study focuses on acute responses to H5N1 virus, other transcriptomic datasets, such as the one reported by Pommerenke and coworkers, encompass acute and adaptive host responses to influenza virus [38]. It will be important to consider changes during adaptive host responses to fully appreciate the impact of magnitude and velocity kinetic effects on the outcome of viral infection.

Several genomic studies have examined host responses to H5N1 virus in mice for a deeper understanding of molecular events impacting viral pathogenesis and dissemination. Cillóniz and coworkers demonstrated robust transcriptional changes of inflammatory response genes, including upregulation of inflammasome genes (e.g., *CASP1*, *IL1B*, and *NLRP3*), in response to H5N1 VN1203 compared to 1918 pandemic influenza virus at day 1 post-infection [6]. Investigation of the host response to viruses possessing specific mutations has also been investigated. For example, Fornek et al. showed that the H5N1 influenza A/Hong Kong/486/97 virus containing amino acid substitution E627K in PB2 upregulated TCR complex genes (e.g., *CD3D* and *CD3G*) in the lungs of infected mice at day 2 post-infection [11]. It must be noted that the PB2 mutation is not the only difference between these two viruses. Our kinetic analysis further supports the likelihood of impaired T cell responses to H5N1 infection. In a separate study, a recombinant virus expressing H5N1 PB1-F2 N66S compared to the wild-type recombinant virus showed differences in the timing of interferon-stimulated gene expression in mouse lung [20]. Proteomic analysis of primary human monocyte-derived macrophages infected with H5N1 virus have also been investigated and changes in protein abundance of components associated with protein synthesis machinery have been observed as early as 1 hour post-infection [39].

Importantly, these kinetic properties of the host response may not be fully captured by traditional statistical methods that largely depend on different assumptions and applied criteria. For instance, methods used for the identification of differentially expressed genes and over-represented pathways can be negatively impacted by 1) gene magnitude changes, via the fold-change cutoff, 2) the dynamics of the host responses, as biological condition comparisons are usually performed within a given time-point, and 3) the variability of the biological conditions, via the p-value cutoff. For example, the comparison of the VN1203 wild-type condition at 10^4^ PFU and at 2 dpi with the VN1203-PB1F2del condition at 10^3^ PFU and at 2 dpi will detect a large number of differentially expressed transcripts or proteins (Figure 5C). On the other hand, a comparison of the VN1203 wide-type condition at 10^4^ PFU and at 2 dpi with the VN1203-PB1F2del condition at 10^3^ PFU and at 2 dpi will detect a smaller number of differentially expressed transcripts and proteins. However, in both cases, the virus triggered the same host response mechanisms, but with different kinetics.

Compare to more traditional analysis methods, the Singular Value Decomposition analysis that we used for the kinetics analysis has several advantages that we exploited here. This analysis is based on an unsupervised method and does not require pulling the samples into distinct groups (i.e. the samples are not grouped by time points post-infection or by inoculation concentrations). This unsupervised aspect was crucial in our analysis because of the shifts on the host response that we described in this study. Using this SVD analysis strategy, we have been able to successfully capture the kinetics pattern of the host response system to VN1203 wild-type without having to group the samples into particular bins. Moreover, the kinetic patterns of the sub-biological mechanisms identified (eigentranscripts and eigenproteins) can be naturally displayed with this analysis strategy. These representations of eigentranscripts and eigenproteins show in a very concise way the kinetics profiles of all the associated transcripts and proteins. For example, we have been able to clearly observe the transient profile of transcripts associated with T-cell responses. With more traditional methods (i.e. gene modules, gene heatmaps or PCA representations in gene space), this kind of representation is not systematic and could produce large unintelligible representations.

The MDS Reference Maps and Projections representations that we developed and used in the context of this study were particularly useful to drive our analysis. By using the VN1203 wild-type virus -omics profiles as a reference representation, we have been able to observe the behavior of the host response to the other variants. Traditional visualization representation methods do not naturally allow fixed positions of a subset of high-dimensional objects. In the context of the development of novel visualization methods [40-42], we showed the relevance of this approach for the analysis of large datasets.

## Conclusions

The emergent properties of a biological system are dynamic and best comprehended by examining the system through the sum of its individual components [43]. Through a systems biology approach, we have demonstrated the importance of the kinetics of the host response to H5N1 pathogenesis. Magnitude and velocity represent emergent properties of the system that are best captured using an expanded time-course, multiple doses and more than one mutant virus. Therefore, changes in gene expression induced by wild-type and mutant virus are not necessarily specific to a particular mutation, but rather encompass kinetic changes where increasing viral dosage is found to proportionally increase the Magnitude Coefficient and impact the timing of peak Velocity Coefficient during infection. Knowledge of the timing of host response changes will have important implications for treatment administration, particularly early in infection.

## Methods

### Viruses

All recombinant influenza viruses – including H1N1 CA04 wild-type, the H5N1 VN1203 wild-type and the panel of four H5N1 VN1203 mutant viruses encoding changes in specific pathogenicity markers – were generated by using reverse genetics coupled with site-directed mutagenesis (for mutant viruses), essentially as described previously [18,44,45].The H5N1 VN1203 mutant viruses included the following: a virus in which the hemagglutinin (HA) surface protein poly-basic cleavage site (RERRRKKR↓G) was mutated to (RETR↓G) (referred to as H5N1 VN1203-HAavir mutant), which has been previously described [2]; a virus possessing a K→E amino acid substitution at residue 627 of the PB2 protein (referred to as H5N1 VN1203-PB2627E), which was previously described [4]; a newly generated virus in which a stop codon mutation was introduced into the NS1 protein open reading frame at amino acid 124, which was achieved by a T→A nucleotide substitution at position 400 of the H5N1 VN1203 NS gene segment (referred to as H5N1 VN1203-NS1trunc); and a newly generated virus in which the PB1-F2 open reading frame was eliminated by introduction of three stop codon mutations (affected nucleotide positions in the H5N1 VN1203 PB1 gene segment: T120C, C153G, G291A; referred to as H5N1 VN1203-PB1F2del). Mutations in the PB1-F2 and NS1 open reading frames did not affect the coding sequences of collinear PB1 and NS2 proteins, respectively. Primer sequences are available upon request.

### Infections and sample collection

All experiments using live H5N1 and H1N1 viruses were performed in an animal enhanced biosafety level 3 (ABSL3 and ABSL3+) containment laboratories at the University of Wisconsin-Madison and University of North Carolina, approved for use by the United States (US) Centers for Disease Control (CDC) and the US Department of Agriculture. All animal experiments and procedures were approved by the University of Wisconsin-Madison School of Veterinary Medicine Animal Care and Use Committee and by the University of North Carolina Institutional Animal Care and Use Committee. Twenty-week-old female C57BL/6J mice (Jackson Laboratories) were anesthetized by isoflurane inhalation or i.p. ketamine/xylazine injection and mock-infected or infected with 10^2^, 10^3^, 10^4^, 10^5^ or 10^6^ Plaque Forming Units (PFU) of virus, as indicated in the text, figures and figure legends. Viruses were diluted in Minimum Essential Medium (MEM) containing 0.3% BSA, and the same medium without virus was used for mock infections. Three to five mice from both mock-infected and infected groups were euthanized at days 1, 2, 4 and 7 post-infection, and lungs were harvested and divided into multiple lobes for virus titration, RNA extraction or protein extraction. For each wild-type or mutant virus, infections were performed in independent experiments, and the same lung lobe was collected for each analysis method from every mouse. Importantly, we did not detect any differences in the amount of virus recovered from any lung lobe, indicating there was no bias in the distribution of viral infection in the mouse lungs (Additional file 8: Figure S5). Individual or group mouse body weights were collected on a daily basis to monitor the disease course, and mice were humanely euthanized upon reaching the experimental endpoint (i.e., sample collection or severe clinical symptoms).

### Median Lethal Dose (MLD) analysis

To determine Median Lethal Dose values (MLD) for VN1203-WT and mutants, we infected 6-week-old BALB/C mice with serial dilutions of each virus (3 mice per dilution) and monitored individual mouse body weight loss and survival over 14 days. All mice were humanely euthanized either when exhibiting severe clinical symptoms, or at the end of the 14 day observation period. MLD values were calculated according to the method of Reed and Muench [46].

### Virus titration

Lung tissues were homogenized using a TissueLyser (Qiagen) or MagnaLyser (Roche) in 1 ml of MEM containing a penicillin/streptomycin mixture (Life Technologies), and following centrifugation to remove debris, virus titrations were performed in Madin-Darby Canine Kidney (MDCK) cells using standard methods. Virus titers are represented as the total number of infectious viruses isolated per lobe.

### Viral mRNA and genomic RNA measurements

Quantification of influenza vRNA and mRNA transcripts was assessed using a strand-specific real-time RT-PCR method previously described [47]. Briefly, H5N1 cDNAs complementary to the two types of influenza viral RNA from the NP genomic segment were synthesized with tagged primers to add an 18–20 nucleotide tag that was unrelated to the influenza virus at the 5’ end (vRNAtag; GGCCGTCATGGTGGCGAAT and mRNAtag; CCAGATCGTTCGAGTCGT). To the mixture, 4 μl of the RNA sample was added with 1.5 μl of 10 μM primer specific for the NP genomic segment vRNA (5'-GGCCGTCATGGTGGCGAATAGCAAAAGCAGGGTAGATAATCACTC-3') or mRNA (5'-CCAGATCGTTCGAGTCGTTTTTTTTTTTTTTTTTCTTTAATTGTC-3') and made up to 13 μl with RNase free water. The mixture was then incubated at 65°C for 5 min and was cooled to 4°C. The reaction mixture [5 μl of First Strand buffer (5×, Invitrogen, Carlsbad, CA), 4 μl of 25 mM MgCl2, 2 μl of 0.1 M dithiothreitol and 1 μl of Superscript II RT (50 U/μl, Invitrogen)] was then added. The RT reaction was carried out at 42°C for 60 min and was terminated by heating at 70°C for 5 min. After the RT reaction, SYBR-Green I real time PCR was then carried out using diluted cDNA as a template, in which 1 μl of cDNA was added to the qPCR reaction mixture [10 μl SYBR GreenERqPCRSuperMix for ABI PRISM (2×), 1.5 μl of each primer for the H5N1 NP genomic vRNA (10 μM; 5'-GGCCGTCATGGTGGCGAAT-3’ and 5'-GTTCCCCACCAGTTTCCATC-3') or mRNA (5'-CCAGATCGTTCGAGTCGT-3' and 5'-CGAACCCGATCGTGCCTTCC-3'), 3 μl of double-distilled water]. The cycle conditions of qPCR were 95°C 10 min, followed by 40 cycles of 95°C 15 s and 60°C for 1 min. H1N1 cDNA was created similarly as above with the NP genomic segment vRNAoligo listed above or mRNA-specific oligo 5’-CCAGATCGTTCGAGTCGTTTTTTTTTTTTTTTTTCAACTGTCATACTC-3’. The genomic vRNA PCR reactions were assembled identically as above for the H5N1 virus, and mRNA reaction was assembled with the mRNA oligo tag primer 5’-CCAGATCGTTCGAGTCG-3’ and H1N1-specific NP mRNA primer 5’-GCTCCCCACCAGTCTCCATT-3’. As an endogenous control, RPL10 mRNA level was measured using primers 5'-TGAAGACATGGTTGCTGAGAAG-3' and 5'-GAACGATTTGGTAGGGTATAGGAG-3'.

### RNA isolation and microarray processing

Lung tissues were directly submerged in RNA-Later stabilization solution (Life Technologies) following dissection, placed at 4°C overnight, followed by freezing at −80°C. Lung tissues were thawed, transferred to 1 ml TRIzol (Life Technologies) and homogenized using a TissueLyser or MagnaLyser. RNA was isolated using QiagenRNeasy Mini columns and the manufacturer’s recommended protocol (Qiagen Inc., Valencia, CA). Fluorescently labeled probes were generated from each RNA sample using Agilent one-color Low Input Quick Amp Labeling Kit (Agilent Technologies). Individual cRNA samples were hybridized to oligonucleotide microarrays for gene expression profiling using Whole Mouse Genome Microarray Kit (Agilent Technologies). All the microarray experiments passed the quality control criteria of the Agilent Feature Extraction Software.

### Transcriptomics data extraction and normalization

Transcriptomics data have been extracted from the AgilentWhole Mouse Genome Microarray raw data and scale normalized [48-50] using the ‘normalizeBetweenArrays’ normalization method available in ‘limma’ package [51] of the R suite [52].

### Proteomics sample preparation

All chemicals and reagents were purchased from Sigma-Aldrich (St. Louis, MO) unless stated otherwise. Ammonium bicarbonate and acetonitrile were purchased from Fisher Scientific (Pittsburgh, PA), and sequencing-grade modified trypsin was purchased from Promega (Madison, WI). Bicinchoninic acid (BCA) assay reagents and standards were obtained from Pierce (Rockford, IL); and purified, deionized water, >18 MΩ, (Nanopure Infinity ultrapure water system, Barnstead, Dubuque, IA) was used to make all aqueous buffers. Dissected lung tissues were directly frozen at −80°C. To prepare protein extracts, thawed tissues were suspended in 1 ml of 8 M urea in 50 mM NH_4_HCO_3_ and homogenized using a TissueLyser or Magnalyser. Protein concentrations of the cleared homogenates were determined by BCA protein assay and diluted to uniform volume in 50 mM ammonium bicarbonate, pH 7.8. Proteins were reduced with 10 mM dithiothreitol, followed by alkylation of free sulfhydryl groups with 40 mM iodoacetamide at 37°C in the dark; each reaction was performed for 1 h at 37°C with constant shaking at 550 rpm. Denatured and reduced samples were diluted 10-fold with 50 mM ammonium bicarbonate, pH 7.8, and CaCl_2_ was added to a final concentration of 1 mM prior to enzymatic digestion. Sequencing-grade modified trypsin was activated by adding 20 μL of 50 mM ammonium bicarbonate, pH 7.8, to 20 μg lyophilized trypsin and incubating for 10 min at 37°C. Activated trypsin was then added to the samples at 1:50 (w/w) trypsin-to-protein ratio, and samples were digested at 37°C for 3 h with constant shaking at 800 rpm; reactions were quenched by rapid freezing in liquid nitrogen. Digested samples were desalted using solid phase extraction columns (Discovery C18, Supelco, Bellefonte, PA), according to the manufacturer’s instructions. Samples were concentrated to 100 μL*in vacuo* (Speed-Vac SC 250 Express, Thermo Savant, Holbrook, NY), and a BCA protein assay was performed to verify final peptide concentrations. Samples were stored at −80°C until strong cation exchange fractionation with liquid chromatography-tandem mass spectrometry (LC-MS/MS) or quantitative LC-MS analyses.

### Strong cation exchange fractionation

Strong cation exchange fractionation was performed as previously described [53]. Twenty four fractions were collected from minute 30 to minute 65 of the gradient, and they were subsequently dried in vacuo and stored at −80°C until LC-MS/MS analysis.

### Reversed-phase capillary LC-MS/MS and LC-MS analyses

Capillary LC-MS/MS analysis was used to generate a peptide accurate mass and time (AMT) tag database [54] for virus-infected lung homogenates (see below). For this, dried peptide fractions were reconstituted in 30 μL of 25 mM ammonium bicarbonate, pH 7.8, and analyzed using a 4-column custom-built capillary LC system coupled online to a linear ion trap mass spectrometer (LTQ; Thermo Scientific, San Jose, CA) by way of an in-house manufactured nanoelectrospray ionization interface, as previously described [55]. To identify the eluting peptides, the LTQ stage was operated in a data-dependent MS/MS mode as previously described [53].

Following lung and virus peptide/protein AMT tag database generation, capillary LC-MS analyses were performed on all individual H5N1 and mock-infected samples to generate quantitative data. For this, dried peptide samples were reconstituted in 30 μL of 25 mM ammonium bicarbonate, pH 7.8, and analyzed in duplicate and random order using identical chromatographic and electrospray conditions as for capillary LC-MS/MS analyses. The LC system was interfaced to an ExactiveOrbitrap mass spectrometer (Thermo Scientific), and the temperature of the heated capillary and the ESI voltage were 250°C and 2.2 kV, respectively. Data were collected over a range of 400–2,000 *m/z*.

### Development of the AMT tag database for virus-infected C57BL/6J mice

An AMT tag database of identified peptides was developed for virus-infected lungs, using mock-infected and virus-infected samples from two studies: 1) H5N1 data reported here and 2) another with SARS-CoV MA15. The details of study 2 will be described elsewhere. To generate the AMT tag database, aliquots of the H5N1- or mock-infected samples were combined to make the following pools: 1) mock-infection (all time points), 2) early infection (1 and 2 d), and 3) late infection (4 and 7 d). Samples from the SARS-CoV MA15 studies were similarly pooled. Each pool was subjected to strong cation exchange fractionation as described above, and each fraction was analyzed by nanocapillary LC-MS/MS. The SEQUEST analysis software [56] was used to match the MS/MS fragmentation spectra with sequences from the April 20, 2010 UniProt/Swiss-Prot rodent protein database, containing 16,244 entries (protein TITIN_Mouse was removed due to excessive length). The data were also matched to sequences from the H5N1 viral proteome. When searching, SEQUEST used a dynamic mass modification on methionine residues corresponding to oxidation (15.99 Da) and a static mass modification on cysteinyl residues to account for alkylation by iodoacetamide (57.02 Da). Peptides passing the following filter criteria were stored as AMT tags in a Microsoft SQL Server database: 1) SEQUEST DelCn2 value (normalized Xcorr difference between the top scoring peptide and the second highest scoring peptide in each MS/MS spectrum) ≥ 0.10 and 2) SEQUEST correlation score (Xcorr) ≥ 1.6, 2.4, and 3.2 for fully tryptic peptides with 1+, 2+, and 3+ charge states, respectively, and Xcorr ≥ 4.3, and 4.7 for partially tryptic or non-tryptic protein terminal peptides with 2+, and 3+ charge states, respectively. Fully non-tryptic peptides were excluded, and a minimum peptide length of 6 amino acid residues was required. These criteria resulted in 39,744 peptides identified with an estimated false discovery rate of <2% based on a target-decoy database search [57]. The elution times for these peptides were normalized to a range of 0 to 1 using a predictive peptide LC normalized elution time (NET) model and linear regression, as previously reported [58]. A NET average and standard deviation were assigned to each identified peptide if the same peptide was observed in multiple analyses. Both calculated monoisotopic masses and observed NETs of identified peptides were included in the AMT tag database. Identified MS/MS spectra corresponding to peptides in the AMT tag database are available at the PRoteomicsIDEntification (PRIDE) database (http://www.ebi.ac.uk/pride/) under the project name A Systems Biology Approach to Emerging Respiratory Viral Diseases in the PRIDE Public Projects folder and corresponding to PRIDE Accession numbers 19855–19860.

### Processing of quantitative LC-MS datasets

Quantitative LC-MS datasets were processed using the PRISM Data Analysis system [59], which is a series of software tools developed in-house (e.g. Decon2LS [60] and VIPER [61]; freely available at http://ncrr.pnl.gov/software/). Individual steps in this data processing approach are reviewed here [54]. The peptide identities for detected features in each dataset (i.e. a single LC-MS analysis) were determined for features matched to AMT tags with high confidence based upon the accurate measured monoisotopic masses and NETs for each of the 39,744 peptides in the filtered AMT tag database within initial search tolerances of ± 6 ppm and ± 0.025 NET for monoisotopic mass and elution time, respectively. The peptides identified from this matching process were retained as a matrix for subsequent data analysis. The raw quantitative proteomics data can be accessed at the PNNL Biological MS Data and Software Distribution Center (http://omics.pnl.gov/) in the Systems Virology Contract Data folder within the Browse Available Data folder.

### Proteomics data processing, missing values imputation, and normalization

Quality control processing was performed to identify and remove contaminant proteins, redundant peptides, peptides with an insufficient amount of data across the set of samples [62], and LC-MS datasets that showed significant deviation from the standard behavior of all LC-MS analyses [63]. The peptide abundance values were normalized across the technical replicates using a global median centering of the data. [64]. The normalized log10 abundance values were averaged across the technical replicates within each biological sample. Peptide level significance patterns were used for protein roll-up to select appropriate peptides for protein quantification. Proteins were quantified using a standard R-Rollup method using the most abundant reference peptide [65], after filtering the peptides that were redundant, had low data content, or were outside the dominant significance pattern.

Missing expression values into each proteomics profile have been inferred using the ‘impute.knn’ k-nearest neighbor imputation method [66] available in the ‘imputation’ package [67] of the R suite [52]. In order to avoid inconsistent missing value imputations, the nearest neighbors have been restricted to proteomics profiles belonging to the same biological condition. The number of nearest neighbors used in the imputation (the k parameter) has been set to 2/3 of the number of samples of the biological condition. Out of the 1,069,500 (3,875x276) proteomic expression values, 450,814 (42.15%) had to be inferred. Biological conditions at the earliest time points post-infection showed the highest degree of imputation, while the biological conditions at the latest time points post-infection showed the lowest degree of imputation. When expression values for a protein having been found as missing in more than 2/3 of the number of samples of the biological condition, the expressions values have been set to 10E-4 in order to avoid infinite fold-changes values when comparing to other conditions.

The whole compendium of proteomics expression profiles center based on the value of the 9^th^ decile and scale normalized [48-50] using the ‘normalizeBetweenArrays’ normalization method available in ‘limma’ package [51] of the R suite [52].

### Identification of differentially expressed transcripts and proteins

Statistically significant differentially expressed transcripts and proteins have been identified using the CDS method [68]. The CDS statistical test allows the identification of differentially but also similarly expressed transcripts and proteins between two biological conditions using a statistical hypothesis test that formulates fold-change statements. We used a fold-change parameter of 2 for the identification of differentially expressed transcripts and a fold-change parameter of 1.2 for the identification of differentially expressed proteins. Transcripts or proteins having a p-value generated by this statistical test lower than 0.01 have been asset as differentially expressed.

### Identification of over-represented biological functions, canonical pathways and upstream regulators

Functional enrichment of biological functions, canonical pathways and upstream regulators was performed using Ingenuity Pathways Analysis (Ingenuity Systems, Inc.). IPA examines differentially expressed transcripts and proteins in the context of known biological functions, mapping each gene identifier to its corresponding molecule in the Ingenuity Pathways Knowledge Base (IPKB). For all analyses, the p-values were generated using the right-tailed Fisher's Exact Test [69].

Upstream Regulator Analysis in IPA incorporates downstream target genes from the experimental dataset and compiled knowledge of reported relationships between regulators and their known target genes within IPKB. This analytical tool was used to predict the upstream regulators in our analysis.

### Multidimensional scaling representations

Multidimensional Scaling representations have been generated using the SVD-MDS method [29]. Multidimensional scaling methods represent the similarities and differences amongst high dimensionality objects into a space having a low number of dimensions, usually in 2 or 3 dimensions for visualization purposes [28]. Pairwise distances between the dots are proportional to the transcriptomic or proteomic distances between the samples. The Kruskal Stress criterion [28] shown in the representations, quantifies the quality of the representations as a fraction of the information lost during the dimensionality reduction procedure. The SVD-MDS method performs the dimensional reduction procedure by using a molecular dynamics approach and by initialing the metric space using a singular value decomposition method, resulting in better representation of the similarities and differences in term of information conservation.

### Identification of transcripts and proteins correlating with the kinetics pattern of the host response to VN1203 wild-type

Transcriptomic and proteomic intensity expression matrices of the wild-type VN1203 infected samples have been factorized using a Singular Value Decomposition method. Transcripts and proteins that correlated with at least one eigentranscript or eigenprotein (right-singular vectors) were then identified using the Pearson’s coefficient of correlation. We used a cutoff of 0.65 on the Pearson’s coefficient of correlation and a cutoff of 0.01 on the p-value associated with this correlation coefficient. The p-values associated with Pearson’s coefficients of correlation have been obtained using the Student's t distribution for a transformation of the correlation. Mock infected biological conditions were not used in the inference of eigentranscripts and eigenproteins, and biological conditions at 1 dpi and 2 dpi were not used in the inference of the eigenproteins because of the relatively low levels of proteins identified as differentially expressed at these time-points. In order to discard transcripts or proteins that could contribute to noise the analysis, only the transcripts and proteins found as differentially expressed in at least one biological condition have been used in the SVD analysis.

The SVD analysis has been performed as described here after. Both the transcriptomic and proteomic expression matrices M (*m×n*, for *m* genes and *n* samples) have been factorized in the form of:

M=USV^t^ where U is a real unitary matrix (*m×m*), S is a rectangular diagonal matrix (*m×n*) with nonnegative real numbers on the diagonal, and V^t^ (the conjugate transpose of V) is a real unitary matrix (*n×n*).

These factorizations have been done using the “svd” function of the R suite [52].

The V matrices have been transposed in order to obtain the eigenvectors (eigentranscripts or eigenproteins). As 52 transcriptomic samples have been used in the SVD factorization, then 52 eigentranscripts have been identified. Respectively, as 22 proteomic samples have been used in the SVD factorization, then 22 eigenproteins have been identified.

Using an iterative procedure, each vector of transcript or protein expression values (of the original expression matrices) have been compared to the identified eigentranscripts or eigenproteins. The statistical comparison has been done using the Pearson’s coefficient of correlation as described before.

We identified a total of 3 eigentranscripts for which at least 5 transcripts were correlating, and we identified a total of 3 eigenproteins for which at least 5 proteins were correlating.

### Magnitude and velocity coefficients

The Magnitude Coefficient (MC) quantifies the magnitude effect as the transcriptomic distance from one biological condition to the matched mock-infected condition. The Velocity Coefficient (VC) quantifies the velocity effect as the speed of the transcriptomic host response to move from one time point to the next one. Both the MC and VC were calculated based on the centroids (arithmetic means) of the biological conditions and the transcriptomic distances (Euclidian distances) are calculated based on the lists of transcripts associated with the kinetics of the host response to VN1203 wild-type. Namely the MC and VC of a biological condition *bc*_*t*_ being the mean vector of gene expression levels amongst the biological replicates at the time point *t* are defined as:

$$\mathrm{MC}\left(bc_{t}\right)=\mathrm{dist}\left(\mathrm{mock},bc_{t}\right)\;\mathrm{and}\;\mathrm{VC}\left(bc_{t}\right)=\frac{\mathrm{dist}\left(bc_{t},bc_{t+1}\right)}{\mathrm{\Delta t}}\;$$

With:

$$\mathrm{dist}\left(c1,c2\right)=\frac{1}{n}\overset{n}{\underset{i}{\sum }}{\left(c1_{i}-c2_{i}\right)}^{2}$$

where: *mock* represents the mean vector of gene expression levels amongst the matched mock-infected samples, Δt is the amount of time between *t* and *t+1* in days, and *n* is the length of the mean vectors of gene expression.

### Multidimensional scaling reference maps and projections

Multidimensional Scaling Reference Maps (MDS Ref Maps) and projections (MDS Projections) have been obtained using a modified version of the SVD-MDS method that we developed and used for this study. While the original SVD-MDS method presented in [29] projects a set of high dimensionality objects into a 2 or 3 dimensional metric space, the modified SVD-MDS method allows to project additional objects over a predefined MDS representation. The predefined MDS representation is named MDS Reference Map, and the overlaid MDS representations are named MDS Projections. The original SVD-MDS method performs the dimensional reduction of the objects by using a molecular dynamics approach, modeling objects by particles and pairwise distances between them by repulsion and attraction forces. The modified SVD-MDS method assigns an infinite mass to each object (i.e. particle) of the MDS Reference Map, resulting in a projection of the additional objects over the predefined representation. The Kruskal Stress criterion [28] shown in the representations quantifies the quality of the representations as a fraction of the information lost during the dimensionality reduction procedure.

### Availability of supporting data

Mice initial body weight loss, viral titer, viral RNA and viral mRNA measurements of the datasets used in this study are available on the Systems Virology website (http://www.systemsvirology.org) on the ‘PROJECT FOLDERS > Home > Data & Resources > Experimental Metadata’. The whole compendium of phenotypical variables measurements used in this study is available under the accession ‘ST003’.

Proteomics processed data are available on the System Virology website (http://www.systemsvirology.org) on the ‘PROJECT FOLDERS > Home > Data & Resources > Proteomic DataPortal’. The normalized expression matrix of the whole compendium of proteomics profiles used in this study is available through the accession id ST003.

Transcriptomic raw data used in this study are available via the following accession identifiers: GSE37569 for the H1N1 CA04 wild-type virus, GSE33263 for the H5N1 VN1203 wild-type virus, GSE37572 for the H5N1 VN1203-HAavir mutant, GSE43301 for the H5N1 VN1203-PB2627E mutant, GSE44445 for the H5N1 VN1203-NS1trunc mutant, GSE43302 for the H5N1 VN1203-PB1F2del mutant at 10E-3 PFU, and GSE44441 for the H5N1 VN1203-PB1F2del mutant at 10E-4 PFU.

## Competing interests

The authors declare that they have no competing interests.

## Authors’ contributions

NT – performed the computational analysis and wrote the manuscript; AJE – contributed to experimental design, data analysis, and manuscript writing; JTG – contributed to data analysis, and manuscript writing; LJ – contributed to data analysis, and manuscript writing; LEG – performed mouse infections using A/CA/04/2009 virus, contributed to experimental design and manuscript editing; BC – contributed to the development of the new geometrical method used to create the MDS Reference Map and MDS Projections design, and manuscript editing; SCT – performed initial proteomic data analysis design, contributed to manuscript editing; BJWR – performed initial proteomic data analysis, contributed to manuscript editing; MTF – titered CA04 samples, contributed to manuscript editing; ALT – performed CA04 mouse infections design, contributed to manuscript editing; CL – performed all infections with VN1203-WT and VN1203 mutant viruses design, titered all VN1203 and mutant lung samples and prepared all VN1203 and mutant lung RNA and protein extractions design, contributed to manuscript editing; GN – contributed to conception and experimental design and manuscript editing; TOM - contributed to conception, experimental design, and manuscript editing and approval; RDS – contributed to conception, experimental design, manuscript editing and approval; KMW– contributed to conception, experimental design, manuscript editing and approval; RB – contributed to conception, experimental design, manuscript editing and approval; YK – contributed to conception, experimental design, manuscript editing and approval; MGK – contributed to conception, experimental design. All authors read and approved the final manuscript.

## Supplementary Material

Additional file 1: Figure S1 Representation of the collected transcriptomic and proteomic profiles of infected mouse lungs along the timeframe. We collected 230 transcriptomic and 198 proteomic profiles of C57BL/6 mouse lung infected by two wild-type (WT) influenza viruses – the H1N1 CA04-WT and the H5N1 VN1203-WT viruses – and 4 mutants of the H5N1 VN1203-WT virus – the H5N1 VN1203-HAavir, H5N1 VN1203-PB2627E, H5N1 VN1203-NS1trunc, and H5N1 VN1203-PB1F2del – at different dosage concentrations (10^2^, 10^3^, 10^4^, 10^5^, and 10^6^ Plaque-Forming Unit – PFU). The transcriptomic and proteomic profiles have been obtained at different days post-infection (1, 2, 4, and 7 days post-infection – dpi). Each dot in the representation represents a transcriptomic profile and each square represents a proteomic profile of a mouse lung sample along the time frame for the different viruses and infection concentrations. Samples of this dataset are gathered into 51 transcriptomic biological conditions and 42 proteomic biological conditions (i.e. set of biological replicates infected by the same virus, at the same infection concentration, and from the same time point post-infection), 13 transcriptomic and 11 proteomic dosage conditions (i.e. set of biological replicates infected by the same virus, and at the same infection concentration), and 6 strain conditions (i.e. set of biological replicate infected by the same virus). Time-matched mock-infected transcriptomic and proteomic profiles have also been collected leading to a total number of 300 transcriptomic and 266 proteomic profiles in our dataset. Click here for file

Additional file 2: Figure S2 Statistical comparison of titer, mRNA and genomic RNA. Graphical table showing the statistical comparisons of viral titer, viral mRNA and viral genomic RNA measurements between all the infection conditions and at all the time points post-infection. The Student's t-test has been used to asset the statistical differences between the groups for all the different kind of measurements. Comparisons having a p-value less than 0.05 have been colored in red. Click here for file

Additional file 3: Table S1 List of transcripts correlating with at least one eigentranscript. For each transcript correlating with at least one eigentranscript identified in the kinetics of host response to H5N1 VN1203 wild-type, the probe identifier, the associated gene name, the associated eigentranscript, and the Pearson’s coefficient of correlation are indicated. Click here for file

Additional file 4: Table S2 List of proteins correlating with at least one eigenproteins. For each protein correlating with at least one eigenprotein identified in the kinetics of host response to H5N1 VN1203 wild-type, the UniProt/SwissProt accession, the associated gene name, the associated eigenprotein, and the Pearson’s coefficient of correlation are indicated. Click here for file

Additional file 5: Table S3 Magnitude and Velocity Coefficients and the phenotypical variables for each transcriptomic biological condition. For each transcriptomic biological condition, the Magnitude Coefficient (MC) – quantifying the Euclidian distance to the matched mock-infected condition – and the Velocity Coefficient (VC) – quantifying the speed of the host response to move to the next time point – are indicated as well as the mean of mice body weight loss (mean body weight) and the difference of the means of viral titer (Δ mean viral titer), viral mRNA (Δ mean viral mRNA), and mean viral genomic RNA (Δ mean viral gRNA) between two consecutive time-points. Click here for file

Additional file 6: Table S3 Heatmap and over-represented pathways of the host response to VN1203-WT and VN1203-NS1trunc at 1 dpi. (A) Heatmap of the transcript expression signals for the VN1203-WT and VN1203-NS1trunc infected transcriptomic profiles at 1 day-post-infection and for the 10^4^ PFU inoculation dosage. Values are shown as log2 ratioed to the mocks infected samples. The dendrogramm representing transcripts clustering has been constructed using the Euclidian metric and complete-link clustering method. (B) Functional enrichment on the 800 transcripts found as over-regulated in VN1203-NS1trunc. 104 significantly over-represented pathways have been identified (p-value cutoff of 0.05) and the top 45 is indicated. For each top over-represented canonical pathway the p-value (shown as -log10) is indicated. Click here for file

Additional file 7: Figure S4 MDS Projection of transcriptomic profiles of the CA04 wild-type infected samples over the VN1203 wild-type MDS Reference Map. (A) Multidimensional Scaling Projection (MDS Projection) of the transcriptomic profiles of the 10^3^ PFU, 10^4^ PFU, 10^5^ PFU and 10^6^ PFU H1N1 CA04 wild-type dosage conditions over the H5N1 VN1203 wild-type Multidimensional Scaling Reference Map (MDS Reference Map). Each dot in the representations is the transcriptomic profile of a biological sample plotted in the intensity space of gene expression. Pairwise distances between the dots are proportional to the transcriptomic distances between the samples. MDS Projections allow to project –omics profiles over a predefined Multidimensional Scaling (MDS) representation. Euclidian distances have been calculated based on the signature of transcripts that significantly correlate with one eigentranscript. Dots are colored in order to indicate the dosage conditions, and biological conditions are indicated by the convex hull of the set of biological replicates (i.e. the smallest convex set containing the points [30]) and labeled to indicate the time point post-infection. Samples and biological conditions of the H5N1 VN1203 wild-type 10^4^ PFU infection dosage are indicated by gray dots and gray convex hulls. Hence the grey spots that are connected represent the transcriptomic profiles of mice lung infected by the VN1203 wild-type virus at 10^4^ PFU, while the ones not connected represent the transcriptomic profiles for the other infection concentrations. The Kruskal Stress shown in each representation quantifies the quality of the geometrical representation as a fraction of the information lost during the dimensionality reduction procedure. Click here for file

Additional file 8: Figure S5 Lung homogeneity. (A) Schematic representation of a lung with the different lobes used for the experiments and measurements. (B) Barplot representation of the viral titer measurements showing that there is no difference in the different lung lobes. Click here for file
