# Supplementary figures and images for: Specific mutations in H5N1 mainly impact the magnitude and velocity of the host response in mice
Source: BMC Syst Biol. 2013 Jul 29;7:69. doi: 10.1186/1752-0509-7-69 (PMC3750405; doi:10.1186/1752-0509-7-69)

# SUPPLEMENTARY FIGURE 1

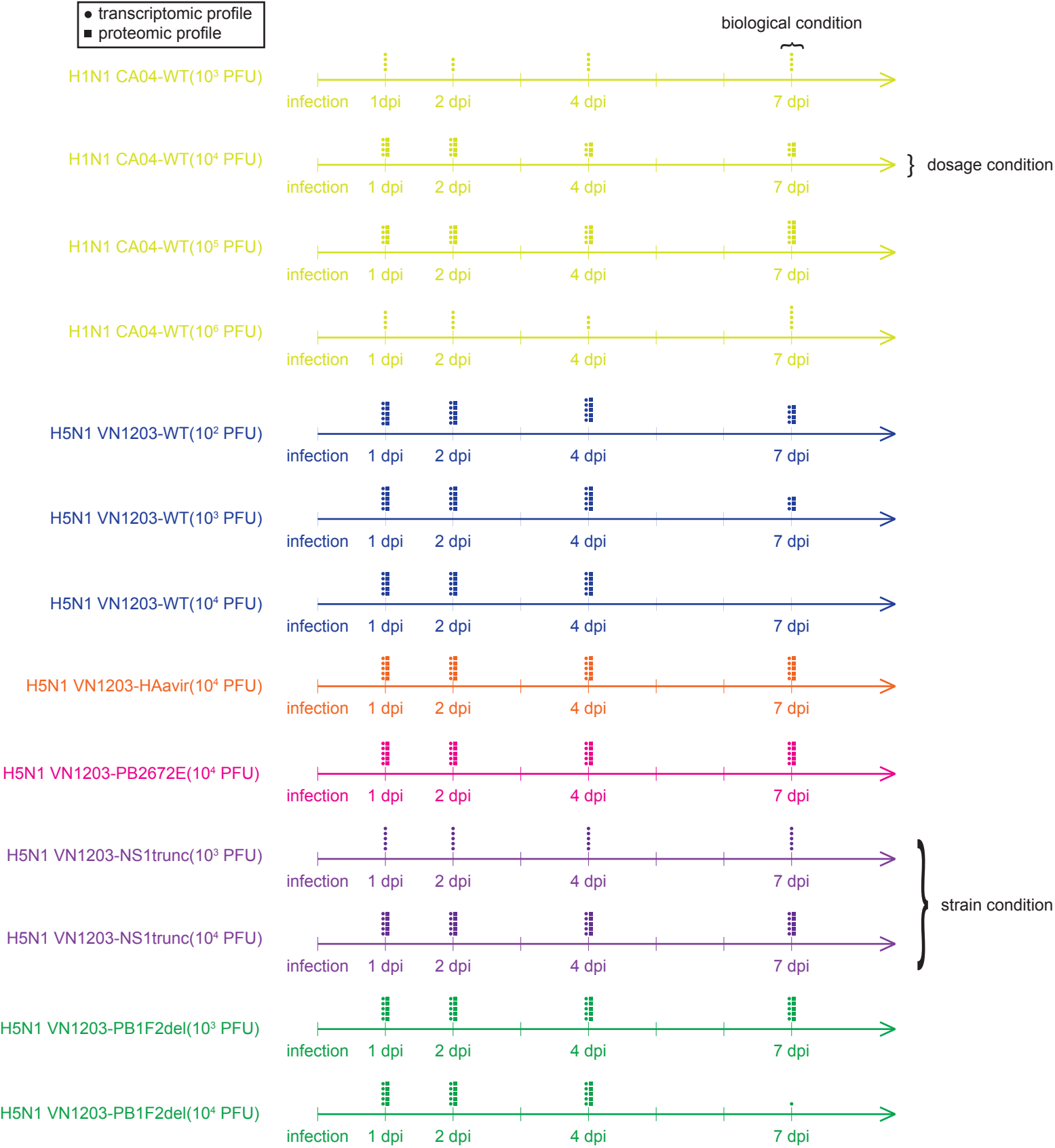

Supplement: Additional file 1: Figure S1 — Representation of the collected transcriptomic and proteomic profiles of infected mouse lungs along the timeframe. We collected 230 transcriptomic and 198 proteomic profiles of C57BL/6 mouse lung infected by two wild-type (WT) influenza viruses – the H1N1 CA04-WT and the H5N1 VN1203-WT viruses – and 4 mutants of the H5N1 VN1203-WT virus – the H5N1 VN1203-HAavir, H5N1 VN1203-PB2627E, H5N1 VN1203-NS1trunc, and H5N1 VN1203-PB1F2del – at different dosage concentrations (102, 103, 104, 105, and 106 Plaque-Forming Unit – PFU). The transcriptomic and proteomic profiles have been obtained at different days post-infection (1, 2, 4, and 7 days post-infection – dpi). Each dot in the representation represents a transcriptomic profile and each square represents a proteomic profile of a mouse lung sample along the time frame for the different viruses and infection concentrations. Samples of this dataset are gathered into 51 transcriptomic biological conditions and 42 proteomic biological conditions (i.e. set of biological replicates infected by the same virus, at the same infection concentration, and from the same time point post-infection), 13 transcriptomic and 11 proteomic dosage conditions (i.e. set of biological replicates infected by the same virus, and at the same infection concentration), and 6 strain conditions (i.e. set of biological replicate infected by the same virus). Time-matched mock-infected transcriptomic and proteomic profiles have also been collected leading to a total number of 300 transcriptomic and 266 proteomic profiles in our dataset. [file 1752-0509-7-69-S1.pdf]

## SUPPLEMENTARY FIGURE 4

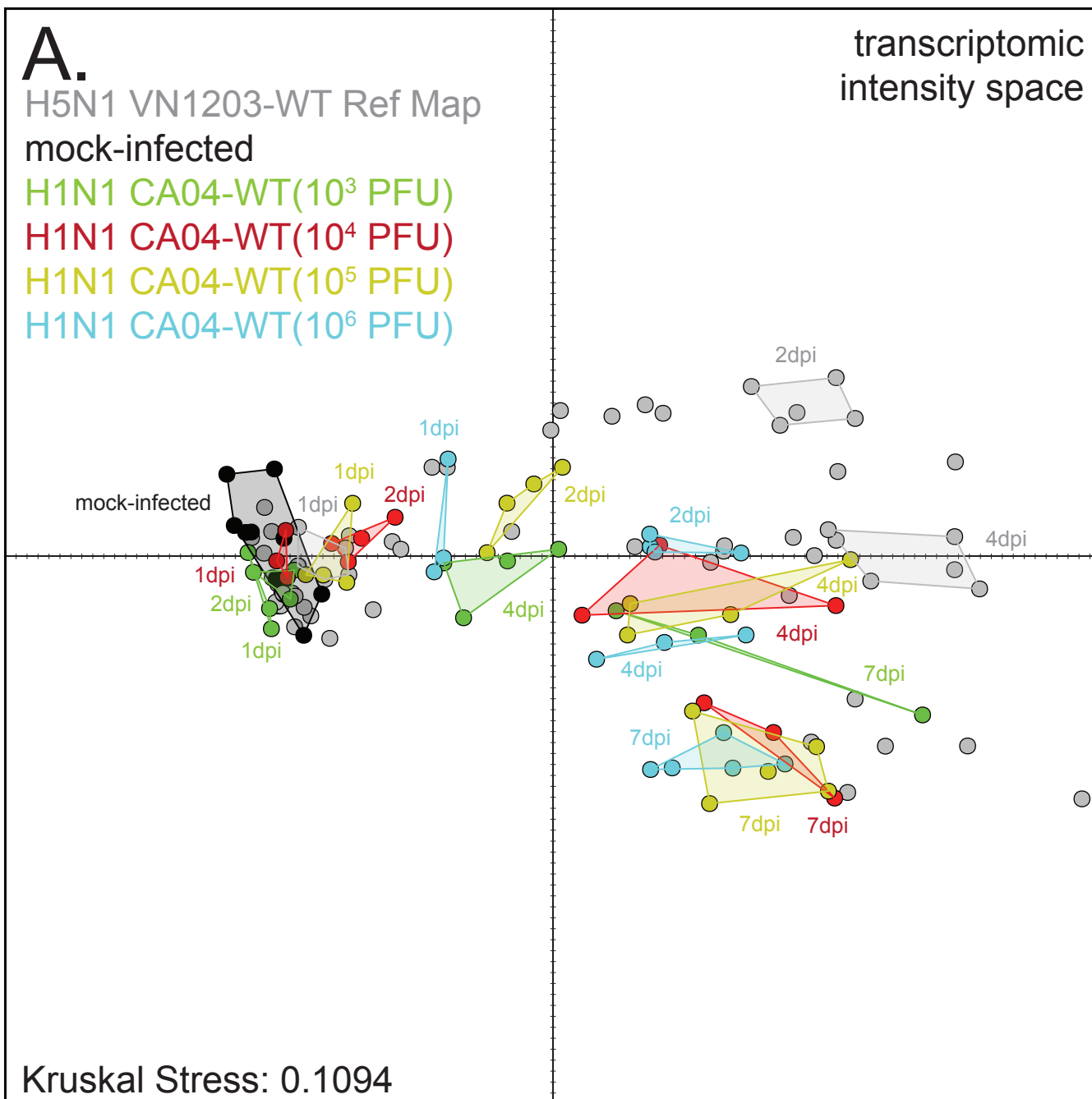

Supplement: Additional file 7: Figure S4 — MDS Projection of transcriptomic profiles of the CA04 wild-type infected samples over the VN1203 wild-type MDS Reference Map. (A) Multidimensional Scaling Projection (MDS Projection) of the transcriptomic profiles of the 103 PFU, 104 PFU, 105 PFU and 106 PFU H1N1 CA04 wild-type dosage conditions over the H5N1 VN1203 wild-type Multidimensional Scaling Reference Map (MDS Reference Map). Each dot in the representations is the transcriptomic profile of a biological sample plotted in the intensity space of gene expression. Pairwise distances between the dots are proportional to the transcriptomic distances between the samples. MDS Projections allow to project –omics profiles over a predefined Multidimensional Scaling (MDS) representation. Euclidian distances have been calculated based on the signature of transcripts that significantly correlate with one eigentranscript. Dots are colored in order to indicate the dosage conditions, and biological conditions are indicated by the convex hull of the set of biological replicates (i.e. the smallest convex set containing the points [30]) and labeled to indicate the time point post-infection. Samples and biological conditions of the H5N1 VN1203 wild-type 104 PFU infection dosage are indicated by gray dots and gray convex hulls. Hence the grey spots that are connected represent the transcriptomic profiles of mice lung infected by the VN1203 wild-type virus at 104 PFU, while the ones not connected represent the transcriptomic profiles for the other infection concentrations. The Kruskal Stress shown in each representation quantifies the quality of the geometrical representation as a fraction of the information lost during the dimensionality reduction procedure. [file 1752-0509-7-69-S7.pdf]
