# Supplementary material for: Specific mutations in H5N1 mainly impact the magnitude and velocity of the host response in mice
Source: BMC Syst Biol. 2013 Jul 29;7:69. doi: 10.1186/1752-0509-7-69 (PMC3750405; doi:10.1186/1752-0509-7-69)
Supplement: Additional file 2: Figure S2 — Statistical comparison of titer, mRNA and genomic RNA. Graphical table showing the statistical comparisons of viral titer, viral mRNA and viral genomic RNA measurements between all the infection conditions and at all the time points post-infection. The Student's t-test has been used to asset the statistical differences between the groups for all the different kind of measurements. Comparisons having a p-value less than 0.05 have been colored in red. [file 1752-0509-7-69-S2.pdf]

SUPLLEMENTARY FIGURE 2

24 h

	CA04	VN1203	HAavir	627E	PB1-F2del	NS1trunc
**TITERS**						
CA04		0.0132	< 0.0001	0.1223	0.1584	0.0157
VN1203			0.0168	0.0058	0.0579	0.0063
HAavir				< 0.0001	0.5394	< 0.0001
627E					0.1146	0.0232
PB1-F2del						0.1315
NS1trunc						
**vRNA**						
CA04		0.0021	0.0005	0.2658	0.0006	0.0006
VN1203			0.1854	0.0006	0.0074	0.0035
HAavir				0.0001	0.0092	0.0022
627E					0.0001	0.0001
PB1-F2del						0.1696
NS1trunc						
**mRNA**						
CA04		0.0027	< 0.0001	0.0386	0.0001	< 0.0001
VN1203			0.2012	0.0006	0.1999	0.169
HAavir				< 0.0001	0.9486	0.7946
627E					< 0.0001	< 0.0001
PB1-F2del						0.8593
NS1trunc						

48 h

	CA04	VN1203	HAavir	627E	PB1-F2del	NS1trunc
**TITERS**						
CA04		0.002	0.001	0.5071	0.0003	0.0003
VN1203			0.0043	0.0006	0.0014	0.0007
HAavir				0.0003	0.0165	0.0004
627E					< 0.0001	0.0026
PB1-F2del						0.0002
NS1trunc						
**vRNA**						
CA04		0.0001	< 0.0001	0.742	0.0161	0.0025
VN1203			0.0072	< 0.0001	0.5603	0.0001
HAavir				< 0.0001	0.2315	< 0.0001
627E					0.0071	0.0235
PB1-F2del						0.0154
NS1trunc						
**mRNA**						
CA04		0.7136	0.063	0.0287	0.0048	0.0369
VN1203			0.0141	< 0.0001	0.0014	0.0052
HAavir				< 0.0001	0.009	0.6788
627E					0.0002	< 0.0001
PB1-F2del						0.0117
NS1trunc						

96 h

	CA04	VN1203	HAavir	627E	PB1-F2del	NS1trunc
**TITERS**						
CA04		0.0303	0.1366	0.203	0.0047	0.0566
VN1203			0.0176	0.0065	0.2773	0.0106
HAavir				0.0951	0.0075	0.4666
627E					0.0005	0.0594
PB1-F2del						0.0017
NS1trunc						
**vRNA**						
CA04		0.0307	0.0139	0.0338	0.001	0.001
VN1203			0.008	0.2245	0.0001	0.0018
HAavir				0.027	0.0325	0.1476
627E					0.0004	0.0545
PB1-F2del						0.0011
NS1trunc						
**mRNA**						
CA04		0.0215	0.2309	0.1625	0.0616	0.0493
VN1203			0.0088	0.0469	< 0.0001	0.0019
HAavir				0.5841	0.0007	0.0046
627E					0.0006	0.0039
PB1-F2del						0.0714
NS1trunc						

168 h

	CA04	VN1203	HAavir	627E	PB1-F2del	NS1trunc
**TITERS**						
CA04			0.4473	0.0521		0.0032
VN1203						
HAavir				0.8208		0.237
627E						0.0086
PB1-F2del						
NS1trunc						
**vRNA**						
CA04			0.0738	0.0372		< 0.0001
VN1203						
HAavir				0.0451		0.9265
627E						< 0.0001
PB1-F2del						
NS1trunc						
**mRNA**						
CA04			0.3226	0.5675		0.0003
VN1203						
HAavir				0.4672		< 0.0001
627E						< 0.0001
PB1-F2del						
NS1trunc						
