# Supplementary material for: Specific mutations in H5N1 mainly impact the magnitude and velocity of the host response in mice
Source: BMC Syst Biol. 2013 Jul 29;7:69. doi: 10.1186/1752-0509-7-69 (PMC3750405; doi:10.1186/1752-0509-7-69)
Supplement: Additional file 6: Table S3 — Heatmap and over-represented pathways of the host response to VN1203-WT and VN1203-NS1trunc at 1 dpi. (A) Heatmap of the transcript expression signals for the VN1203-WT and VN1203-NS1trunc infected transcriptomic profiles at 1 day-post-infection and for the 104 PFU inoculation dosage. Values are shown as log2 ratioed to the mocks infected samples. The dendrogramm representing transcripts clustering has been constructed using the Euclidian metric and complete-link clustering method. (B) Functional enrichment on the 800 transcripts found as over-regulated in VN1203-NS1trunc. 104 significantly over-represented pathways have been identified (p-value cutoff of 0.05) and the top 45 is indicated. For each top over-represented canonical pathway the p-value (shown as -log10) is indicated. [file 1752-0509-7-69-S6.pdf]

SUPLLEMENTARY FIGURE 3

A.

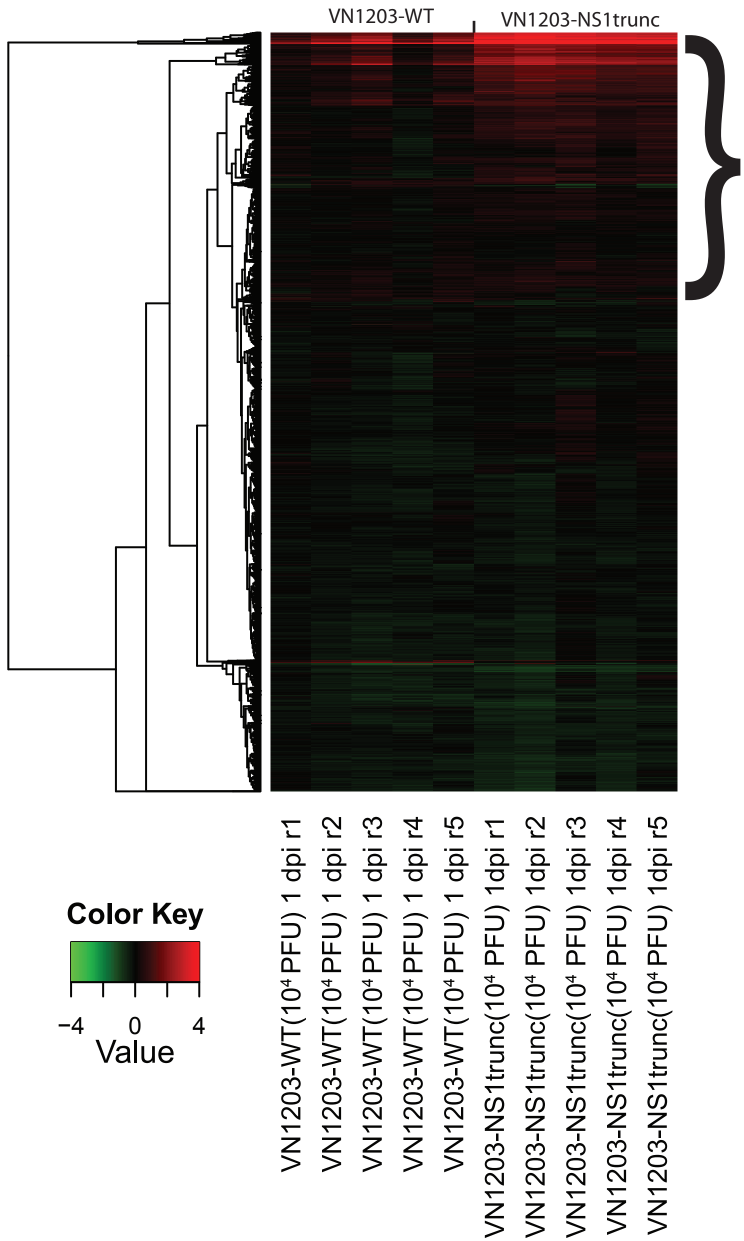

B.

Functional Enrichement

Canonical Pathways	-log(p-value)
Role of Pattern Recognition Receptors in Recognition of Bacteria and Viruses	1.28E+01
Interferon Signaling	1.19E+01
Activation of IRF by Cytosolic Pattern Recognition Receptors	1.15E+01
Communication between Innate and Adaptive Immune Cells	1.09E+01
TREM1 Signaling	9.96E+00
Antigen Presentation Pathway	9.58E+00
Crosstalk between Dendritic Cells and Natural Killer Cells	9.23E+00
Dendritic Cell Maturation	8.31E+00
Type I Diabetes Mellitus Signaling	7.58E+00
Pathogenesis of Multiple Sclerosis	7.56E+00
Allograft Rejection Signaling	7.49E+00
Acute Phase Response Signaling	7.40E+00
OX40 Signaling Pathway	6.95E+00
Role of Macrophages, Fibroblasts and Endothelial Cells in Rheumatoid Arthritis	6.73E+00
Cytotoxic T Lymphocyte-mediated Apoptosis of Target Cells	6.46E+00
Atherosclerosis Signaling	6.28E+00
Role of RIG1-like Receptors in Antiviral Innate Immunity	6.20E+00
IL-17A Signaling in Fibroblasts	6.08E+00
Hepatic Fibrosis / Hepatic Stellate Cell Activation	5.76E+00
Role of Hypercytokinemia/hyperchemokinemia in the Pathogenesis of Influenza	5.49E+00
Role of PKR in Interferon Induction and Antiviral Response	5.44E+00
Role of IL-17A in Arthritis	5.24E+00
Differential Regulation of Cytokine Production in Macrophages and T Helper Cells by IL-17A and IL-17F	5.20E+00
Role of IL-17F in Allergic Inflammatory Airway Diseases	5.16E+00
NF-ÎºB Signaling	4.95E+00
Retinoic acid Mediated Apoptosis Signaling	4.91E+00
TNFR2 Signaling	4.67E+00
IL-10 Signaling	4.37E+00
Altered T Cell and B Cell Signaling in Rheumatoid Arthritis	4.34E+00
Role of JAK1, JAK2 and TYK2 in Interferon Signaling	4.30E+00
TWEAK Signaling	4.17E+00
LXR/RXR Activation	4.15E+00
Death Receptor Signaling	4.13E+00
IL-17 Signaling	4.09E+00
Neuroprotective Role of THOP1 in Alzheimer's Disease	4.00E+00
TNFR1 Signaling	3.97E+00
IL-15 Production	3.96E+00
IL-17A Signaling in Gastric Cells	3.96E+00
Role of JAK family kinases in IL-6-type Cytokine Signaling	3.96E+00
Production of Nitric Oxide and Reactive Oxygen Species in Macrophages	3.92E+00
IL-17A Signaling in Airway Cells	3.84E+00
Graft-versus-Host Disease Signaling	3.68E+00
Complement System	3.67E+00
Role of PI3K/AKT Signaling in the Pathogenesis of Influenza	3.65E+00
iNOS Signaling	3.54E+00
