# Supplementary material for: Specific mutations in H5N1 mainly impact the magnitude and velocity of the host response in mice
Source: BMC Syst Biol. 2013 Jul 29;7:69. doi: 10.1186/1752-0509-7-69 (PMC3750405; doi:10.1186/1752-0509-7-69)

SUPLLEMENTARY FIGURE 5

A.

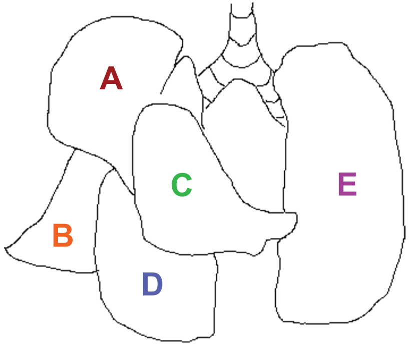

Sample Collection Experiments:

A/C - RNA (microarray)

B - Proteins (global proteomics)

D - Virus titer

E - Histology

B.

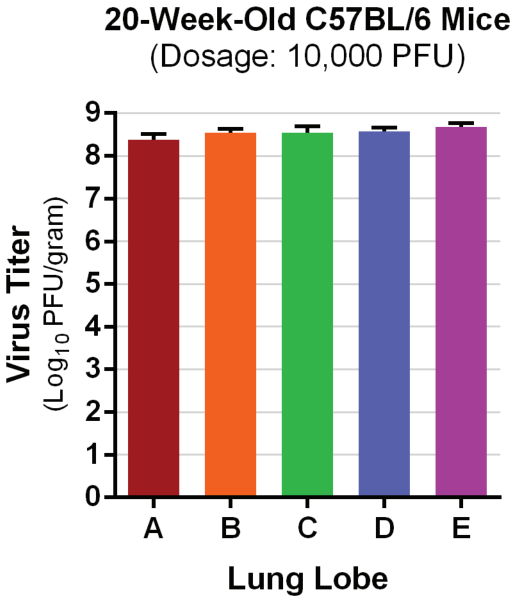

Supplement: Additional file 8: Figure S5 — Lung homogeneity. (A) Schematic representation of a lung with the different lobes used for the experiments and measurements. (B) Barplot representation of the viral titer measurements showing that there is no difference in the different lung lobes. [file 1752-0509-7-69-S8.pdf]
